# VRK1/BANF1/GLI1 Axis Regulates Tumor Development and Progression of Colorectal Cancer

**DOI:** 10.7150/ijbs.107279

**Published:** 2025-04-28

**Authors:** Xu Wang, Yuanmin Xu, Shixin Chan, Hu Zhao, Siwei Huang, Yiming Wang, Yang Yang, Zhenglin Wang, Xiaomin Zuo, Huabing Zhang, Wei Chen

**Affiliations:** 1 Department of General Surgery, The First Affiliated Hospital of Anhui Medical University, Hefei 230032, Anhui, China.; 2 Department of Biochemistry and Molecular Biology, Metabolic Disease Research Center, School of Basic Medicine, Anhui Medical University, Hefei 230032, Anhui, China.

**Keywords:** CRC, BANF1, prognosis, GLI1, VRK1

## Abstract

**Rationale:** The association between Barrier to Autointegration Factor 1 (BANF1) and various human diseases has been recently reported. However, its role and mechanism in colorectal cancer (CRC) initiation and progression remain unexplored.

**Methods:** This study examined BANF1 expression in CRC tissues and cells using bioinformatics databases, PCR, Western Blot (WB), and immunohistochemistry (IHC). The role of BANF1 in the initiation and progression of CRC was evaluated through both *in vitro* and *in vivo* experiments. RNA sequencing was employed to explore potential mechanisms, which were subsequently validated experimentally. Furthermore, a database-driven approach predicted an upstream protein interacting with BANF1, and its role in CRC was validated.

**Results:** BANF1 expression was found to be elevated in both CRC cell lines and tissues, establishing BANF1 as an independent prognostic factor for CRC patients. Experiments conducted both *in vitro* and *in vivo* revealed that BANF1 influences CRC phenotypes through the regulation of GLI1 expression. Bioinformatics analyses predicted an interaction between BANF1 and vaccinia-related kinase 1 (VRK1), which was confirmed through functional validation. VRK1 was identified as an upstream regulator of BANF1, interacting with it at the protein level to influence CRC phenotypes.

**Conclusion:** The study offers insights into CRC's molecular mechanisms and proposes targeting the VRK1/BANF1/GLI1 axis as a potential therapeutic strategy. This method could result in more effective treatments for advanced CRC.

## Introduction

Colorectal cancer (CRC) poses a significant threat to public health worldwide, ranking third in incidence and second in mortality among all cancers 1. Global cancer statistics predict that by 2030, CRC incidence will reach approximately 2.2 million cases, with over 1.1 million associated deaths 2. In China, cancer surveys reveal a yearly increase in CRC incidence, with CRC ranking fourth in incidence and fifth in cancer-related mortality 3. Despite extensive research, the etiology of CRC remains incompletely understood. Evidence-based medicine suggests that CRC may be closely linked to factors such as genetic mutations, high-fat diets, chronic inflammation, immune response dysregulation, and intestinal microbiota dysbiosis 4. The TNM staging system categorizes CRC by assessing tumor invasion depth, lymph node involvement, and distant metastasis, which collectively determine the overall stage from I to IV 5. CRC staging correlates strongly with the five-year survival rates of patients. Survival rates are significantly higher for patients with early-stage CRC than for those with advanced-stage disease. The five-year survival rates for CRC are about 90% for stage I, between 63% and 87% for stage II, and from 53% to 89% for stage III 6. Patients with distant metastases exhibit a five-year survival rate of merely 14% 7. Early symptoms of CRC are often nonspecific and overlooked, leading to advanced-stage diagnoses in many cases. Even after primary lesion resection, tumor recurrence occurs in 30-50% of cases 8. Improving treatment efficacy for advanced-stage CRC remains a significant challenge for researchers.

The development of CRC is a multistep, multistage, and multigene process in which normal colonic epithelial cells progress to colorectal adenomas that may eventually lead to CRC. This progression is associated with mutations in various genes and the loss of DNA mismatch repair capability 9. Researchers identified several oncogenic pathways, including KRAS, BRAF, PTEN, IGFR1, and EGFR, along with epigenetic alterations like DNA methylation dysregulation, chromatin remodeling, and miRNA-mediated gene expression control, a decade ago 10-13. In recent years, advances in targeted therapies and the application of immunotherapy have expanded treatment options for patients with CRC. Immune therapy targeting programmed death receptor-1 has shown positive outcomes in metastatic CRC patients with mismatch repair deficiency or high microsatellite instability 14. These treatments are effective for only a limited group of CRC patients, highlighting the critical need for new early diagnostic markers and therapeutic targets. Such developments could provide new treatment approaches and improve prognostic predictions for patients with CRC.

Barrier to Autointegration Factor 1 (BANF1) is a DNA-binding protein that anchors DNA to nuclear membrane structural proteins 15. BANF1 participates in multiple biological processes such as mitosis, nuclear assembly, viral infection, chromatin and gene regulation, and the DNA damage response 16. BANF1 regulates DNA-dependent protein kinase (DNA-PK) activity, affecting the double-strand DNA break repair pathway in DNA damage repair 17. BANF1 modulates poly (ADP-ribose) polymerase 1 (PARP1) activity post-oxidative DNA damage by directly interacting with DNA repair proteins, initiating the repair process 18. BANF1 is crucial in the context of inflammatory bowel disease. A recent study 19 found that knocking out Transmembrane and immunoglobulin domain-containing protein 1 (TMIGD1) impairs intestinal barrier integrity in Crohn's disease. TMIGD1 directly interacts with BANF1, inhibiting NF-κB pathway activation and leading to intestinal inflammation. The exogenous expression of TMIGD1 and BANF1 enhances intestinal barrier function and reduces inflammation in both *in vitro* and *in vivo* models. Numerous studies indicate a strong association between BANF1 and the development and advancement of various tumors. BANF1 expression is notably higher in triple-negative breast cancer tissues than in normal breast tissues, showing a strong correlation with lymph node metastasis and TNM staging 20. In cervical cancer, BANF1 knockdown significantly inhibits tumor cell clonogenicity, invasion, and migration, underscoring its role in cervical cancer progression 21. Similarly, Li *et al.* 22 investigated the expression levels of BANF1 and its upstream regulator vaccinia-related kinase 1 (VRK1) in esophageal squamous cell carcinoma tissues. The study demonstrated elevated mRNA and protein expression levels of BANF1 and VRK1 in tumor tissues relative to adjacent normal tissues. ROC curve analysis identified VRK1 and BANF1 as potential therapeutic targets for esophageal squamous cell carcinoma. Although BANF1 is linked to the development and progression of various cancers, its role and mechanisms in CRC have not been investigated.

This study examined BANF1 expression in CRC cell lines and tissues, performing statistical analyses to evaluate its clinical significance in relation to expression levels and clinical data. Experiments conducted both *in vitro* and *in vivo* revealed that BANF1 affects CRC phenotypes through the regulation of GLI1 expression. Additionally, bioinformatics approaches predicted an interaction between BANF1 and VRK1. Subsequent analyses explored the phenotypic and functional implications of this interaction, providing evidence that VRK1 acts as an upstream regulator of BANF1, with a possible interaction at the protein level.

## Materials and methods

### Tissue collection

Sixty-eight pairs of CRC tissues were obtained from surgical samples at the First Affiliated Hospital of Anhui Medical University from January 1, 2014, to December 31, 2016, for tissue microarray construction, IHC staining, and follow-up analyses. Ten pairs of tumor and adjacent normal tissues were also collected for Western blot analysis. Samples were stored at -80°C until use. The Ethics Committee of the First Affiliated Hospital of Anhui Medical University approved this study, and all patients provided informed consent. The surgical samples used in this research were confirmed as CRC based on postoperative pathology reports. Prior to surgery, no patients underwent neoadjuvant chemotherapy, radiotherapy, or any pharmacological treatments.

### RNA extraction and qRT-PCR

RNA was isolated following the manufacturer's instructions using an extraction kit. RNA quality and quantity were evaluated with a spectrophotometer. RNA was converted to cDNA using a reverse transcription kit following the manufacturer's guidelines. qRT-PCR was performed with specific primers for target genes and SYBR Green PCR master mix. Reactions were conducted in a 96-well plate with cDNA as the template. Target gene expression levels were normalized to glyceraldehyde-3-phosphate dehydrogenase using the 2-ΔΔCT method. Primer sequences for PCR amplification are provided in Supplementary Table S1.

### Protein extraction and WB

Cells were collected and lysed using RIPA buffer (Beyotime, China) with added protease and phosphatase inhibitors. The lysate was chilled on ice before centrifugation to obtain the supernatant. Protein concentration was measured using a bicinchoninic acid assay kit according to the manufacturer's guidelines. Proteins were combined with loading buffer in equal proportions and denatured. The samples were loaded onto a 10% polyacrylamide gel for electrophoresis. Proteins were transferred to a polyvinylidene fluoride membrane via the wet transfer method. The membrane was blocked in blocking buffer and probed with primary antibodies against the target proteins, diluted in blocking buffer, and incubated overnight. An HRP-conjugated secondary antibody was applied following the washing step. Protein bands were visualized using an enhanced chemiluminescence detection system, and images were captured with a gel documentation system. Antibodies used in this study are listed in Supplementary Table S2.

### IHC

Slides underwent deparaffinization in xylene followed by rehydration through a series of graded alcohol concentrations. Slides underwent antigen retrieval through microwave heating in citrate buffer, then cooled to room temperature. Endogenous peroxidase activity was inhibited with 3% hydrogen peroxide. The slides were blocked with bovine serum albumin prepared in phosphate-buffered saline (PBS). Sections were incubated overnight at 4°C with primary antibodies in blocking buffer. Post-washing, the sections were treated with a biotinylated secondary antibody and then an avidin-biotin complex reagent. Color development utilized a 3,3'-diaminobenzidine substrate. Sections were counterstained with hematoxylin, dehydrated through graded alcohols, and mounted using a suitable mounting medium. Images of the slides were captured using a light microscope.

### Hematoxylin and Eosin (HE) staining

Paraffin-embedded tissue sections were placed in an oven for gradient deparaffinization, followed by washes in absolute ethanol. The sections were stained with hematoxylin and subsequently blued in warm water. Nuclear staining was examined under a microscope. The sections were stained with eosin solution, rinsed in tap water for 1 minute, and examined microscopically for cytoplasmic staining. Following drying, sections were mounted using neutral resin and then observed and photographed under a microscope for analysis.

### Co-Immunoprecipitation

Total protein was extracted by harvesting and lysing cells with a lysis buffer containing protease inhibitors. The cell lysate was pre-cleared by incubating with protein A/G beads, followed by their removal through centrifugation. The pre-cleared lysate was incubated overnight with a specific antibody targeting the protein of interest. The mixture was incubated with Protein A/G beads to capture immune complexes. The beads underwent centrifugation and were washed thrice with wash buffer to eliminate non-specifically bound proteins. Bound proteins were eluted using elution buffer containing high salt concentrations. Eluted proteins underwent SDS-PAGE and subsequent Western Blot analysis to verify protein-protein interactions.

### Immunofluorescence staining

Slides were deparaffinized using xylene and rehydrated with graded alcohols. Sections underwent antigen retrieval through microwave heating in citrate buffer, then cooled to room temperature. The sections underwent permeabilization using 0.1% Triton X-100, followed by PBS washing. Slides were incubated with blocking solution to minimize non-specific binding. The sections were incubated overnight with primary antibodies in a blocking solution. Following incubation, sections were rinsed thrice with PBS and then incubated in the dark with fluorophore-conjugated secondary antibodies. After the final wash, coverslips were mounted with a DAPI-containing medium for nuclear staining. Fluorescence images were captured using a fluorescence microscope.

### Cell culture and transfection

Cells were maintained in DMEM with 10% fetal bovine serum and 1% penicillin-streptomycin, incubated at 37°C in a 5% CO₂ environment. Cells were passaged biennially. Cells at 70-80% confluence were trypsinized, resuspended in fresh medium, and seeded in 6-well plates at 3 × 10^5^ cells per well. The cells were allowed to adhere for 24 hours prior to transfection. Plasmid DNA was transfected using Lipofectamine 3000, following the manufacturer's guidelines. The cells were incubated with the mixture for 24-48 hours. After transfection, the medium was substituted with a puromycin-containing selective medium to isolate successfully transfected cells. Selection was sustained for 1-2 weeks, with the medium refreshed every 2-3 days. Surviving colonies were picked and expanded in selective medium. Small interfering RNA (siRNA) used in this study was purchased from Tsingke Biotechnology Co., Ltd. (China), and overexpression plasmids were obtained from Unibio (China). The sequences of siRNAs are provided in Supplementary Table S3.

### CCK-8 assay

A 96-well plate was seeded with cells at a density of 3000 per well. After adherence, the cells were treated with various concentrations of test compounds or control treatments and incubated for the desired time period under standard conditions. After treatment, 10 μL of CCK-8 solution (Dojindo Molecular Technologies) was added to each well, followed by incubation for 1 hour. A microplate reader measured absorbance at 450 nm, which directly correlated with the number of viable cells.

### Colony formation assay

Single-cell suspensions were prepared, and 1000 cells were seeded into each well of a 6-well plate containing complete growth medium. Cells were incubated for 10-14 days to facilitate colony formation. The medium was replaced every 3-4 days to maintain optimal growth conditions. After colony formation, the medium was discarded, and the cells were carefully rinsed with PBS. Colonies were fixed with 4% paraformaldehyde and subsequently stained with crystal violet solution. Colonies containing at least 50 cells were counted.

### Transwell assay

Transwell inserts were placed into the wells of a 24-well plate. For invasion assays, the insert membrane's upper surface could be coated with Matrigel. A cell suspension in serum-free medium was placed in the upper chamber of each insert, with the lower chamber containing complete medium with 10% FBS serving as a chemoattractant. The plate was incubated for 24-48 hours, allowing cells to migrate or invade through the membrane pores toward the lower chamber. Cells that migrated to the membrane's lower surface were fixed using 4% paraformaldehyde and stained with crystal violet. The membrane was washed, dried, and observed under a microscope.

### Wound healing assay

Cells were seeded into a 6-well plate at a density sufficient to reach 90-100% confluence within 24 hours. The cells were incubated to achieve a confluent monolayer. A sterile 200 μL pipette tip was employed to create a linear scratch on the confluent cell monolayer, simulating a wound. The wells were carefully rinsed with PBS to eliminate detached cells and debris from the scratched region. The scratch area was imaged immediately after its creation and at regular intervals using a phase-contrast microscope.

### Assessment of radiosensitivity

1,000 cells were seeded into six-well plates and allowed to adhere before further processing. Cells were exposed to a single irradiation session using 6-MeV X-rays at a dose rate of 300 cGy/min, with doses of 0, 2, 4, and 6 Gy, and a source-to-surface distance of 100 cm. The plate surfaces were covered with 1 cm of tissue-equivalent material to ensure uniform exposure. Following irradiation, the cells were cultured for approximately 10 days to allow visible colony formation. After discarding the culture medium, cells were washed thrice with PBS, fixed in 4% formaldehyde, and stained with crystal violet. Colony counts were assessed using Image J software, and the relative survival rate was computed as the survival fraction, defined by the formula: (colonies in the experimental group / colonies in the control group) × 100%.

### Xenograft tumor growth model

HCT-116 cells were collected in the exponential growth phase, quantified, and resuspended in a PBS and Matrigel mixture. Nude mice, aged four weeks, were kept in pathogen-free conditions. Animal experiments adhered to institutional ethical guidelines and received ethics committee approval. A suspension containing approximately 5 × 10⁶ cells in 100 μL was subcutaneously injected into the flanks of nude mice using a sterile 1 mL syringe. Bilateral injections were performed when appropriate to accommodate multiple experimental conditions. Tumor progression was assessed every 3-4 days by measuring its length and width using calipers. Once tumors reached the maximum allowable size, the mice were humanely euthanized. Tumors were excised, weighed, and subjected to further analyses, including histological examination, IHC, and molecular assays.

### Clinical data

We used the Kaplan-Meier method and the log-rank test to assess overall survival differences between groups with high and low BANF1 expression. Cox regression analyses, both univariate and multivariate, were performed to assess the influence of BANF1 expression levels and other clinical variables such as age, sex, carcinoembryonic antigen (CEA) levels, and AJCC staging. Factors significantly linked to patient prognosis in univariate or multivariate analyses were used to develop a nomogram model. The nomogram, constructed from multivariate Cox regression coefficients, was validated for predictive accuracy using a calibration curve. To enhance the clinical applicability of the prognostic model, we created a web-based prognostic calculator for patients with CRC using R and deployed it via the shinyapps platform to generate an accessible webpage link. Clinical information for patients with CRC is provided in Supplementary Table S4.

### Bioinformatic analyses

Transcriptomic data for pan-cancer were obtained from the TCGA and GTEx databases. Eight single-cell datasets (EMTAB8107, GSE103224, GSE159115, GSE166635, EMTAB6149, GSE162708, and GSE176031) were obtained from the Tumor Immune Single-cell Hub 2 (TISCH2) portal (http://tisch.comp-genomics.org/home/). Statistical analysis of differential BANF1 expression across pan-cancer types was performed using the Wilcoxon test and paired-sample t-test. Visualization was conducted using the R packages* gganatogram* and *ggplot2*. The diagnostic significance of BANF1 in colon and rectum adenocarcinomas was evaluated through the ROC curve's area under the curve analysis. Expression differences in paired and unpaired samples of BANF1 in TCGA-CRC dataset were analyzed and visualized using the Xiantao platform (https://www.xiantaozi.com/). Cell annotation for the single-cell datasets was accomplished using the *singleR* package, followed by visualization of BANF1 expression across different single-cell datasets using R software.

### Flow cytometry assay

Cells were treated with trypsin and then resuspended in complete medium. The cell suspension was collected in a centrifuge tube and centrifuged at 1000 × g for 5 minutes. The supernatant was carefully discarded to avoid aspirating the cells. The cells were washed at least twice with pre-chilled PBS. Binding Buffer was diluted 1:4 with deionized water. Cells were resuspended in 250 μL of Binding Buffer to achieve a concentration of 1 × 10⁶ cells/mL. A 100 μL cell suspension was placed in a 5 mL flow cytometry tube, followed by the addition of 5 μL Annexin V-PE and 10 μL 7-AAD solution. The mixture was gently stirred and left to incubate in the dark at room temperature for 15 minutes. Following incubation, 400 μL of PBS was added to the reaction tube for analysis via flow cytometry.

### RNA-sequencing

The HCT116 cell line with BANF1 knockdown (n = 3) and the control cell line (n = 3) were used for RNA-seq. RNA extraction was performed using TRIzol reagent. Transcriptome sequencing and data analysis were conducted by GENE DINOVO Corporation.

### Statistical analyses

Statistical analyses were conducted using GraphPad Prism 9.4.1 or R 4.3.3, with results presented as mean ± standard deviation (SD). Group differences were evaluated using the Student's t-test or one-way ANOVA, considering a *p*-value < 0.05 as statistically significant.

## Results

### Expression of BANF1 in various malignant tumors

**Figure 1:**
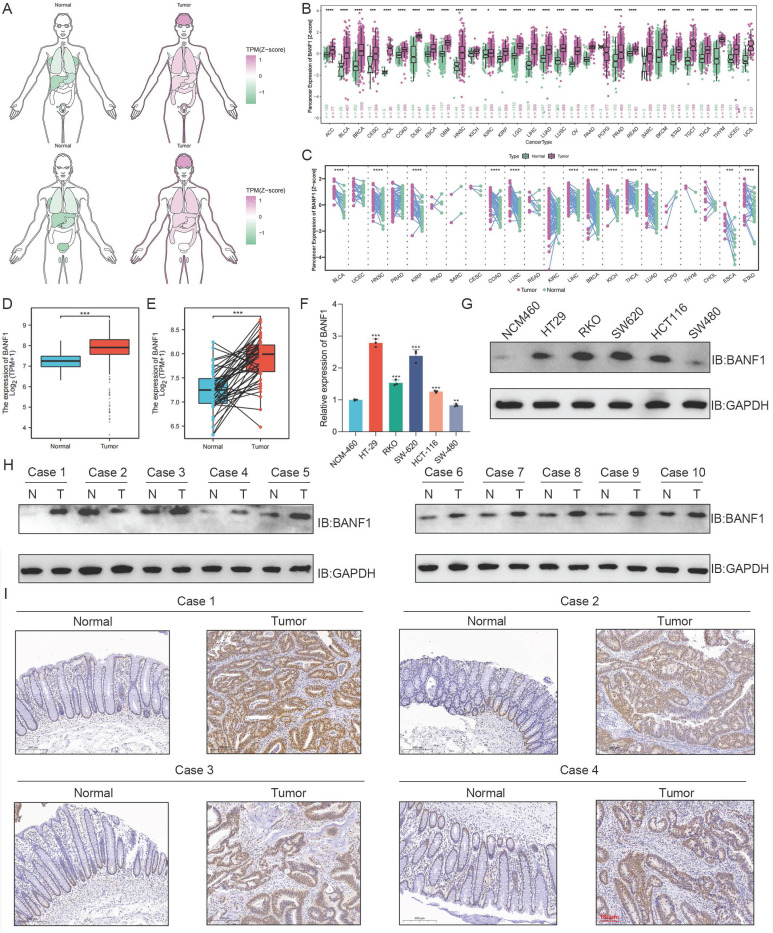
**Expression of BANF1 is elevated in CRC cell lines and tissues.** (A) BANF1 expression levels are significantly elevated in various malignancies. BANF1 expression is markedly increased across multiple malignancies in both unpaired (B) and paired samples (C) (*p* < 0.001). (D-E) BANF1 mRNA expression levels are significantly elevated in CRC tissues from the TCGA database. (F) Results of qRT-PCR showed that mRNA expression of BANF1 is higher in most CRC cell lines compared with NCM-460. (G) Protein expression levels were shown using WB experiment. (H) The majority of CRC tissues exhibited significantly elevated BANF1 expression (I) the staining intensity for the BANF1 antibody was notably greater in most CRC patient tissues. *p < 0.05; **p < 0.01; ***p < 0.001; ****p < 0.0001.

Compared to normal tissues and organs, BANF1 expression levels are significantly elevated in various malignancies (Figure 1A). BANF1 expression is markedly increased across multiple malignancies in both unpaired (Figure 1B) and paired samples (Figure 1C) (*p* < 0.001). In unpaired samples, BANF1 is notably upregulated in COAD and READ. BANF1 is broadly expressed in various cell types, including epithelial, tumor, and immune cells, across multiple malignant tumors such as invasive breast cancer, colorectal adenocarcinoma, glioma, clear cell renal carcinoma, hepatocellular carcinoma, non-small cell lung cancer, pancreatic cancer, and prostate cancer (Supplementary Figure S1A-H).

### BANF1 is significantly overexpressed in CRC and correlates with patient outcome

**Figure 2:**
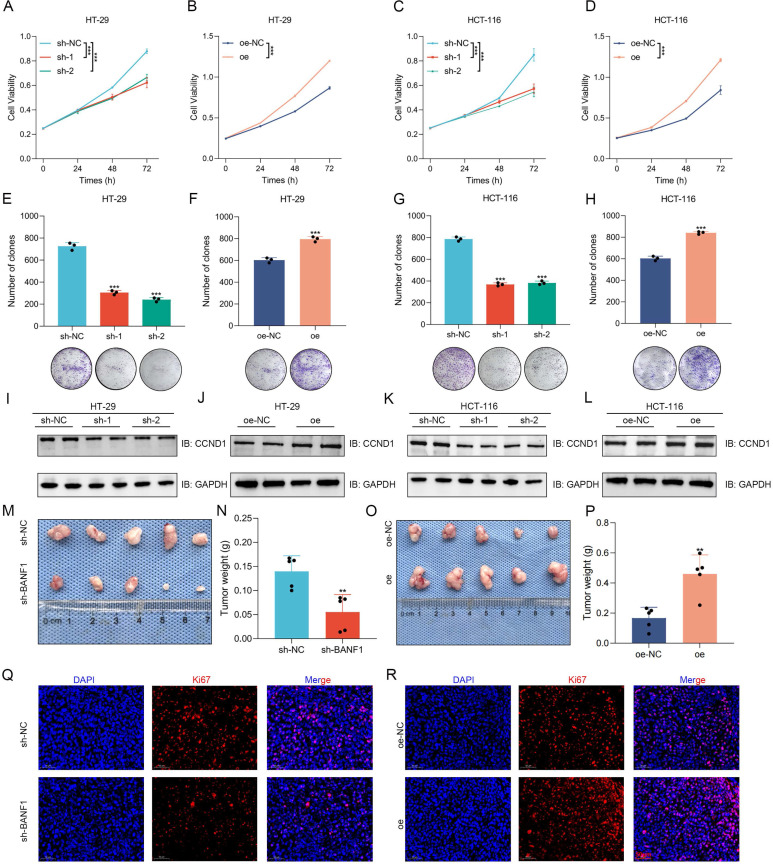
** BANF1 exerts significant effects on the proliferation of CRC both *in vitro* and *in vivo*.** (A-D) CCK-8 assay indicate that silencing BANF1 significantly inhibited cell proliferation in both HT-29 and HCT-116 cell lines, overexpression of BANF1 markedly enhanced cell proliferation. (E,G) Silencing BANF1 led to a substantial reduction in the clonogenic capacity of both CRC cell lines. (F,H) Overexpression of BANF1 significantly increased the clonogenic potential. (I,K) WB analysis revealed a decrease in the protein expression level of CCND1 following the silencing of BANF1. (J, L) In cells overexpressing BANF1, the protein expression of CCND1 was significantly elevated. (M-N) Silencing BANF1 in CRC cells led to significantly smaller subcutaneous tumors and decreased tumor weights compared to the control group. (P-O) Overexpressing BANF1 led to significantly larger subcutaneous tumors and increased tumor weights. (Q-R) Following the knockdown of BANF1, the intensity of Ki67 staining was markedly reduced. In contrast, overexpression of BANF1 resulted in increased Ki67 levels within the tumors. **p < 0.01; ***p < 0.001.

BANF1 mRNA expression levels are significantly elevated in CRC tissues from the TCGA database (Figure 1D-E). Compared to the NCM-460 cell line, BANF1 expression is markedly increased in HT-29, RKO, SW-620, and HCT116 cell lines (*p* < 0.001), while expression levels in the SW-480 cell line are lower than those in NCM-460 (*p* < 0.01) (Figure 1F). WB analysis revealed that BANF1 expression was higher in HT-29, RKO, SW-620, and HCT116 cell lines compared to NCM-460, whereas SW-480 showed a slight decrease in expression (Figure 1G, Supplementary Figure S2A). Most CRC tissues exhibited significantly elevated BANF1 expression (Figure 1H, Supplementary Figure S2B). Staining intensity for the BANF1 antibody was notably greater in CRC tissues than in normal tissues (Figure 2I, Supplementary Figure S2C). Patients were categorized into high-BANF1 (n = 37) and low-BANF1 (n = 31) expression groups based on IHC staining scores. Univariate (Supplementary Figure S3A) and multivariate (Supplementary Figure S3B) Cox regression analyses demonstrated that BANF1 is a robust predictor of overall survival. Integrating BANF1 expression with other critical clinical indicators, including age, preoperative CEA levels, and AJCC staging, a nomogram model was developed (Supplementary Figure S3C). The nomogram's predictive accuracy was evaluated using a calibration plot (Supplementary Figure S3D). Kaplan-Meier survival analysis revealed that CRC patients with low BANF1 expression exhibited significantly improved prognoses and extended survival durations compared to those with high expression levels (*p* = 0.013, Supplementary Figure S3E). To facilitate clinical application, an online web calculator (https://doctorwang.shinyapps.io/BANF1/, Supplementary Figure S4) was developed. This tool provides valuable insights for clinical practice and supports treatment decision-making.

### BANF1 exerts significant effects on the proliferation of CRC both *in vitro* and *in vivo*

The efficiency of BANF1 knockdown and overexpression was validated through WB analysis (Supplementary Figure S5A-D). The CCK-8 assay results indicated that BANF1 silencing markedly reduced cell proliferation in HT-29 and HCT-116 cell lines (Figure 2A-D). Conversely, BANF1 overexpression markedly enhanced cell proliferation. Silencing BANF1 led to a substantial reduction in the clonogenic capacity of both CRC cell lines, resulting in fewer colonies (Figure 2E, Figure 2G). In contrast, overexpression of BANF1 significantly increased the clonogenic potential, with a notable rise in colony numbers (Figure 2F, Figure 2H). Western blot analysis showed reduced cyclin D1 (CCND1) protein expression after BANF1 silencing. In cells overexpressing BANF1, CCND1 protein levels were significantly elevated (Figure 2I-L, Supplementary Figure S5E-H). In subcutaneous tumor models using nude mice, BANF1 silencing in CRC cells led to significantly smaller tumors and reduced tumor weight compared to controls (*p* < 0.01) (Figure 2M-N, Supplementary Figure S6A). In contrast, cells overexpressing BANF1 produced larger tumors than those in the overexpression negative control (oe-NC) group (Figure 2O, Supplementary Figure S6D). Measurements of tumor volume (Supplementary Figure S6B, E) and body weight (Supplementary Figure S6C, F) were recorded every four days, showing increased tumor weight as well (*p* < 0.01) (Figure 2P). Following BANF1 knockdown, Ki67 staining intensity was markedly reduced, indicating lower Ki67 expression. Conversely, overexpression of BANF1 resulted in increased Ki67 levels in tumors (Figure 2Q-R, Supplementary Figure S6G-H). IHC and HE staining analyses of BANF1 in subcutaneous tumor models using nude mice are shown in Supplementary Figure S7A-B.

### BANF1 influences CRC metastasis *in vitro* and *in vivo*

**Figure 3:**
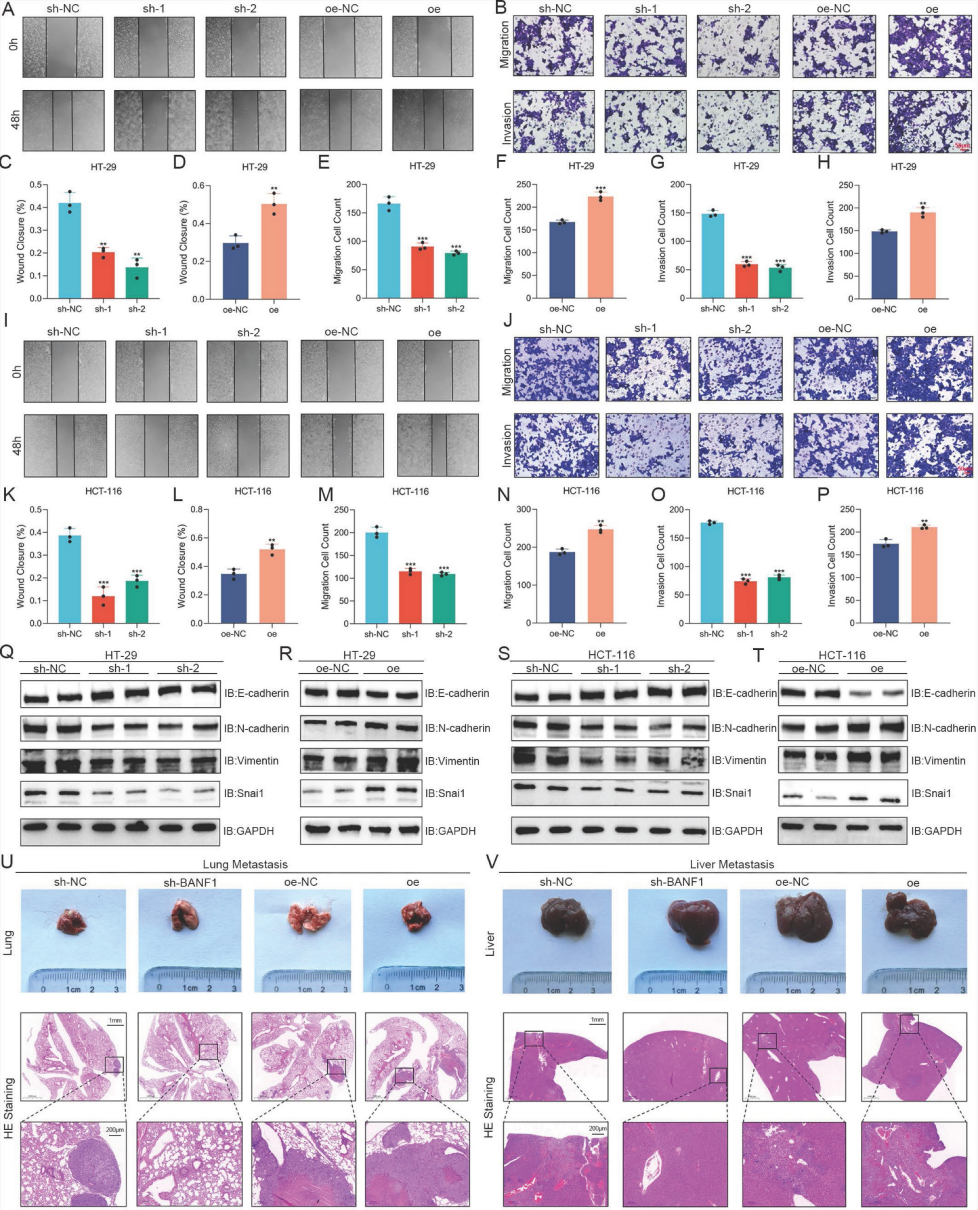
** BANF1 influences CRC metastasis *in vitro* and *in vivo*.** (A-H) Wound healing and Transwell assays demonstrated a significant reduction in the migratory capacity of HT-29 cells following the knockdown of BANF1, while overexpression of BANF1 led to a marked increase in cell migration and invasion. (I-P) Consistent results were observed in HCT-116 cells, where knockdown and overexpression of BANF1 significantly inhibited and promoted the migratory and invasive capabilities. (Q-T) In HT-29 and HCT-116 cell lines, BANF1 knockdown exhibited increased expression of E-cadherin and decreased levels of N-cadherin, Vimentin, and Snai1 proteins, overexpressing BANF1 reduced E-cadherin expression, while levels of N-cadherin, Vimentin, and Snai1 proteins were significantly elevated. (U-V) Mice injected with BANF1 knockdown cells exhibited a significantly reduced number of lung or liver metastatic nodules, while those injected with BANF1 overexpressing cells developed a greater number of metastases. **p < 0.01; ***p < 0.001.

Wound healing assays showed a significant reduction in HT-29 cell migration following BANF1 knockdown, while BANF1 overexpression markedly enhanced migration (Figure 3A, C-D). Similarly, Transwell assays indicated that BANF1 modulates migration and invasion in HT-29 cells (Figure 3B, E-H). Consistent results were observed in HCT-116 cells, where BANF1 knockdown and overexpression inhibited and promoted migratory and invasive capabilities, respectively (Figure 3I-P). In HT-29 cells, silencing BANF1 elevated E-cadherin levels while reducing the expression of N-cadherin, Vimentin, and Snai1 (Figure 3Q, Supplementary Figure S8A). Conversely, BANF1 overexpression reduced E-cadherin levels while elevating N-cadherin, Vimentin, and Snai1 expression (Figure 3R, Supplementary Figure S8B). Similar trends were observed in HCT-116 cells (Figure 3S-T, Supplementary Figure S8C-D). Mouse models of lung and liver metastasis were established via tail vein injection of tumor cells in nude mice. Examination of lung and liver tissues, along with HE staining, revealed that BANF1 knockdown significantly reduced metastatic nodules, while BANF1 overexpression increased metastasis (Figure 3U-V).

### BANF1 regulates apoptosis of CRC *in vitro* and *in vivo*

**Figure 4:**
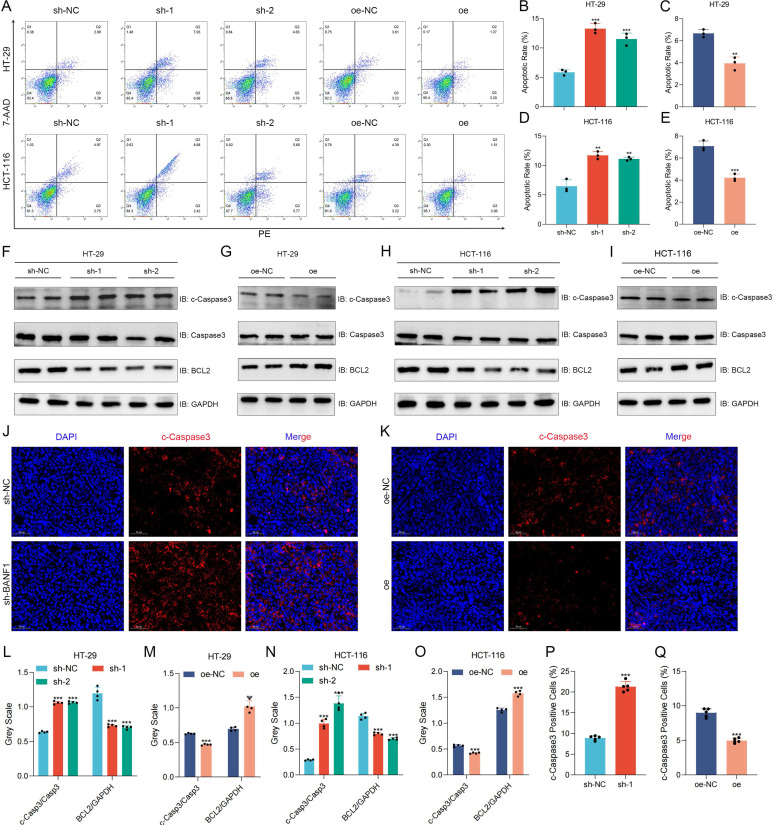
** BANF1 regulates apoptosis of CRC *in vitro* and *in vivo*.** (A-E) Knockdown of BANF1 resulted in a significantly lower apoptosis rate in HT-29 and HCT-116 cells compared to the control group, overexpression of BANF1 led to a marked decrease in the apoptosis rates. (F-I) Knockdown of BANF1 led to an increase in the expression of cleaved-caspase-3 protein compared to total caspase-3, while BCL2 protein levels decreased, overexpression of BANF1 resulted in a decrease in cleaved-caspase-3 expression and an increase in BCL2 levels. (J-K) The tumors from the BANF1 knockdown group exhibited higher cleaved-caspase-3 staining intensity, the oe group showed decreased cleaved-caspase-3 staining intensity compared to the oe-NC group. **p < 0.01; ***p < 0.001.

BANF1 knockdown significantly increased apoptosis rates in HT-29 and HCT-116 cells relative to the control group. Overexpression of BANF1 significantly reduced apoptosis rates in both cell lines relative to the oe-NC group (Figure 4A-E). BANF1 knockdown in both cell lines resulted in elevated cleaved-caspase-3 expression compared to total caspase-3, alongside reduced BCL2 protein levels (Figure 4F, H, L, N). Overexpression of BANF1 decreased cleaved-caspase-3 expression and increased BCL2 levels relative to the control group (Figure 4G, I, M, O). Tumors from the BANF1 knockdown group exhibited higher cleaved-caspase-3 staining intensity, indicating increased apoptosis among tumor cells (Figure 4J, P). Conversely, tumors from the BANF1 overexpression group showed reduced cleaved-caspase-3 staining intensity compared to the oe-NC group (Figure 4K, O).

### Knockdown and overexpression of BANF1 affect the sensitivity of CRC cells to radiotherapy

As radiation dose increased, levels of γ-H2AX protein in HT-29 and HCT-116 cells also rose accordingly (Supplementary Figure S9A-B; Supplementary Figure S10A-B). In HT-29 cells, BANF1 knockdown groups exhibited significantly reduced colony survival rates compared to the sh-NC group at irradiation doses of 2 Gy, 4 Gy, and 6 Gy. Colony survival rates were significantly higher in the BANF1 overexpression group compared to the oe-NC group (Supplementary Figure S9C-E). In HCT-116 cells, BANF1 knockdown increased radiotherapy sensitivity, while its overexpression decreased it (Supplementary Figure S9F-H). In the HT-29 and HCT-116 cell lines, sh-1 and sh-2 groups exhibited significantly reduced expression levels of DNA-PKcs, KU70, KU80, and RAD51 compared to the sh-NC group (Supplementary Figures S9I, K; Supplementary Figure S10C, E). The BANF1 overexpression group showed significantly higher levels of DNA-PKcs, KU70, KU80, and RAD51 compared to the oe-NC group. These findings indicate that BANF1 knockdown reduces the DNA damage repair capacity of CRC cells, while BANF1 overexpression enhances this repair capability (Supplementary Figure S9J, L; Supplementary Figure S10D, F).

### Analysis of BANF1-related functions and pathways using RNA-Seq and experimental validation

**Figure 5:**
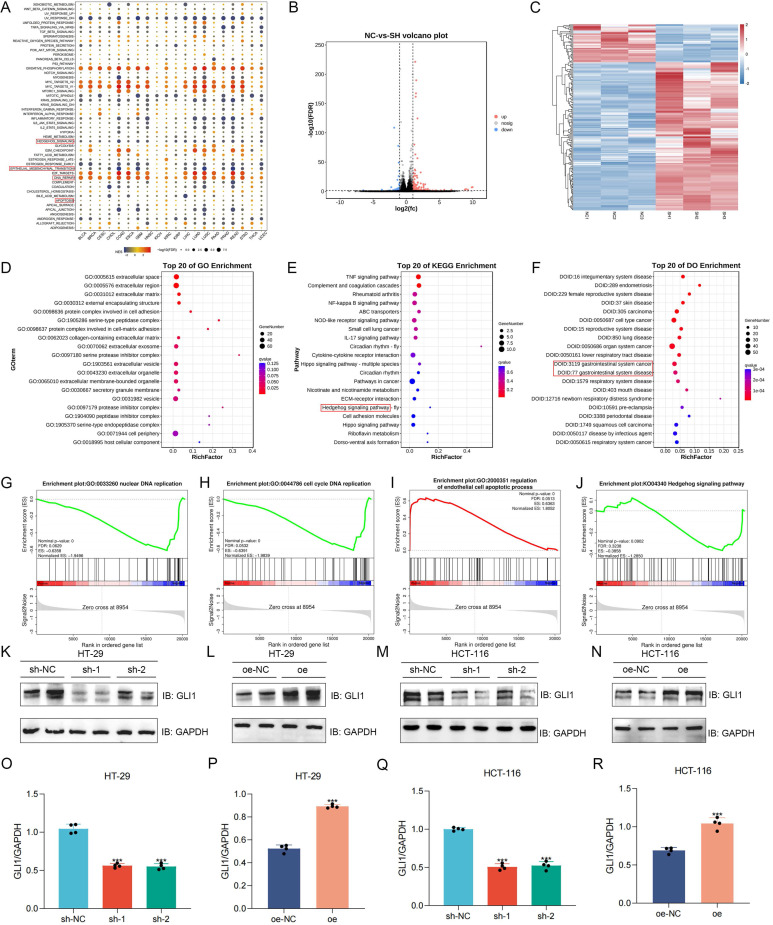
** Analysis of BANF1-related functions and pathways using RNA-Seq and experimental validation.** (A) The results of GSEA indicated that BANF1 is associated with the Hedgehog pathway, EMT, DNA repair, and apoptosis. (B-C) Transcriptome sequencing was performed on HCT-116 cells with sh-NC and sh-1 (n=3), generating volcano plots and heatmaps of differentially expressed genes. (D) GO analysis revealed that BANF1 is primarily related to extracellular matrix components. (E) KEGG analysis indicated associations with the TNF signaling pathway, NF-kappa B signaling pathway, Hippo signaling pathway, and Hedgehog signaling pathway. (F) DO analysis demonstrated that BANF1 correlates with numerous human diseases, including gastrointestinal disorders and malignant tumors. (G-J) In HCT-116 cells with BANF1 knockdown, nuclear DNA replication and cell cycle DNA replication were inhibited, while the endothelial cell apoptosis process was upregulated, and the Hedgehog signaling pathway was downregulated. (K-N) GLI1 protein expression was down-regulated or increased after knocking down or overexpressing BANF1. ***p < 0.001.

Gene Set Enrichment Analysis (GSEA) of pan-cancer data identified BANF1's association with the Hedgehog pathway, epithelial-mesenchymal transition (EMT), DNA repair, and apoptosis in COAD and READ (Figure 5A). Transcriptome sequencing was performed on HCT-116 cells with sh-NC and sh-1 (n = 3), generating volcano plots (Figure 5B) and heatmaps (Figure 5C) of differentially expressed genes. Gene Ontology (GO) analysis revealed BANF1's primary association with extracellular matrix components (Figure 5D). Concurrently, Kyoto Encyclopedia of Genes and Genomes (KEGG) analysis identified its correlation with the TNF, NF-kappa B, Hippo, and Hedgehog signaling pathways (Figure 5E). Disease Ontology (DO) analysis demonstrated that BANF1 is linked to various human diseases, including gastrointestinal disorders and malignant tumors (Figure 5F). GSEA of transcriptome data revealed that, in HCT-116 cells with BANF1 knockdown, nuclear DNA replication (Figure 5G) and cell cycle DNA replication (Figure 5H) were inhibited, while endothelial cell apoptosis was upregulated (Figure 5I), and the Hedgehog signaling pathway was downregulated (Figure 5J). qRT-PCR analysis of three Hedgehog pathway genes, GLI1, SHH, and SMO, showed significantly reduced mRNA expression levels of GLI1 and SMO after BANF1 knockdown in HCT-116 cells (Supplementary Figure S11A-C). Furthermore, we assessed protein level changes in Hedgehog pathway components SMO (Supplementary Figure S12A-D) and GLI1 following BANF1 knockdown and overexpression using WB analysis. In HT-29 cells, GLI1 protein levels were significantly reduced in the sh-1 and sh-2 groups compared to the sh-NC group (Figure 5K), while overexpression of BANF1 led to increased GLI1 protein expression relative to the oe-NC group (Figure 5L). In HCT-116 cells, BANF1 knockdown reduced GLI1 protein levels compared to the control group (Figure 5M), whereas BANF1 overexpression increased GLI1 protein levels (Figure 5N). A quantitative assessment of WB results for GLI1 in BANF1 knockdown and overexpression cell lines was also conducted (Figure 5O-R).

### Overexpression or knockdown of GLI1 can restore the effect of BANF1 on the proliferation and migration of CRC cells

**Figure 6:**
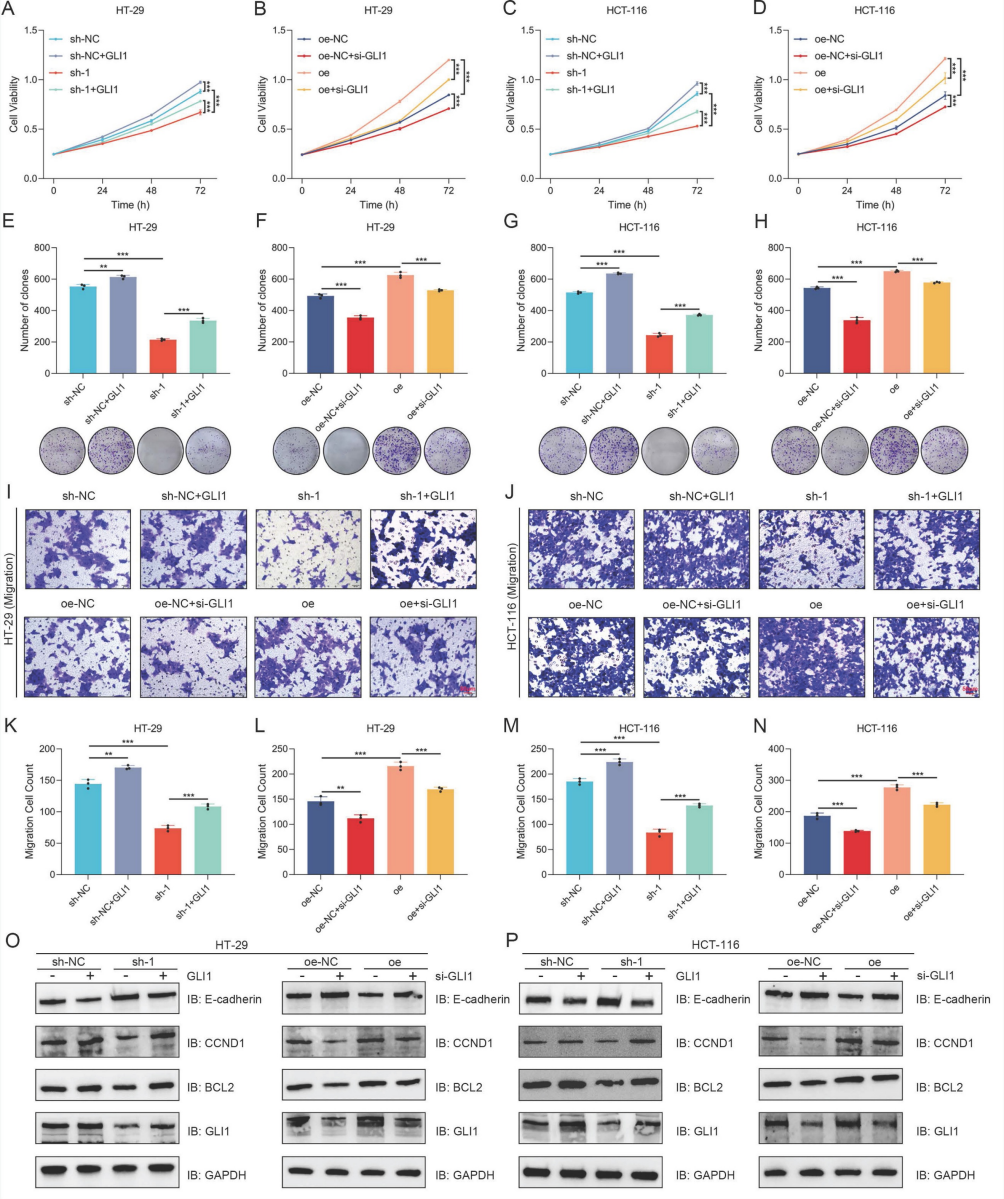
** Overexpression or knockdown of GLI1 can restore the effect of BANF1 on the proliferation and migration of CRC cells.** (A-B, E-F) Following the transfection of a GLI1 overexpression plasmid, control and sh-1 cells exhibited enhanced proliferative activity and increased clonogenic potential. (C-D, G-H) After transfection with si-GLI1, both the oe-NC and oe groups showed a significant reduction in cell viability and clonogenic formation. (I-N) The reduced migratory ability of cells upon BANF1 knockdown and the increased migratory capacity following BANF1 overexpression could both be reversed by the upregulation or downregulation of GLI1. (O-P) Upon transfection with a GLI1 overexpression plasmid, the protein levels of GLI1, BCL2, and CCND1 were significantly elevated in both the sh-NC and sh-1 groups of both cell lines, while the protein level of E-cadherin was reduced. Conversely, after treatment with si-GLI1, the protein levels of GLI1, BCL2, and CCND1 were markedly decreased in the oe-NC and oe groups, whereas E-cadherin levels were significantly increased. **p < 0.01; ***p < 0.001.

Following the transfection of a GLI1 overexpression plasmid, both control and sh-1 cells exhibited enhanced proliferative activity and increased clonogenic potential (Figure 6A-B, Figure 6E-F). Following si-GLI1 introduction, HT-29 and HCT-116 cells in the oe-NC and oe groups exhibited notably decreased cell viability and clonogenic formation (Figure 6C-D, Figure 6G-H). Transwell assay results indicated that GLI1 modulation could reverse the effects of BANF1 expression changes on cell migration, with GLI1 upregulation or downregulation counteracting the reduced migration from BANF1 knockdown and the enhanced migration from BANF1 overexpression. Statistical analysis revealed that transfection with a GLI1 overexpression plasmid enhanced migratory potential in control and sh-1 cells. Following si-GLI1 introduction, cell migration was notably decreased in both the oe-NC and oe groups of HT-29 and HCT-116 cells (Figure 6I-N). Western blot analysis indicated that transfecting the GLI1 overexpression plasmid notably increased GLI1, BCL2, and CCND1 protein levels, while decreasing E-cadherin protein levels in both sh-NC and sh-1 groups across the cell lines. Conversely, si-GLI1 treatment markedly decreased GLI1, BCL2, and CCND1 protein levels in the oe-NC and oe groups, while significantly increasing E-cadherin levels (Figure 6O-P). Quantitative analysis of the WB results from the rescue experiments is provided in Supplementary Figure S13A-D.

### Protein interaction prediction analysis suggested that VRK1 may interact with BANF1

**Figure 7:**
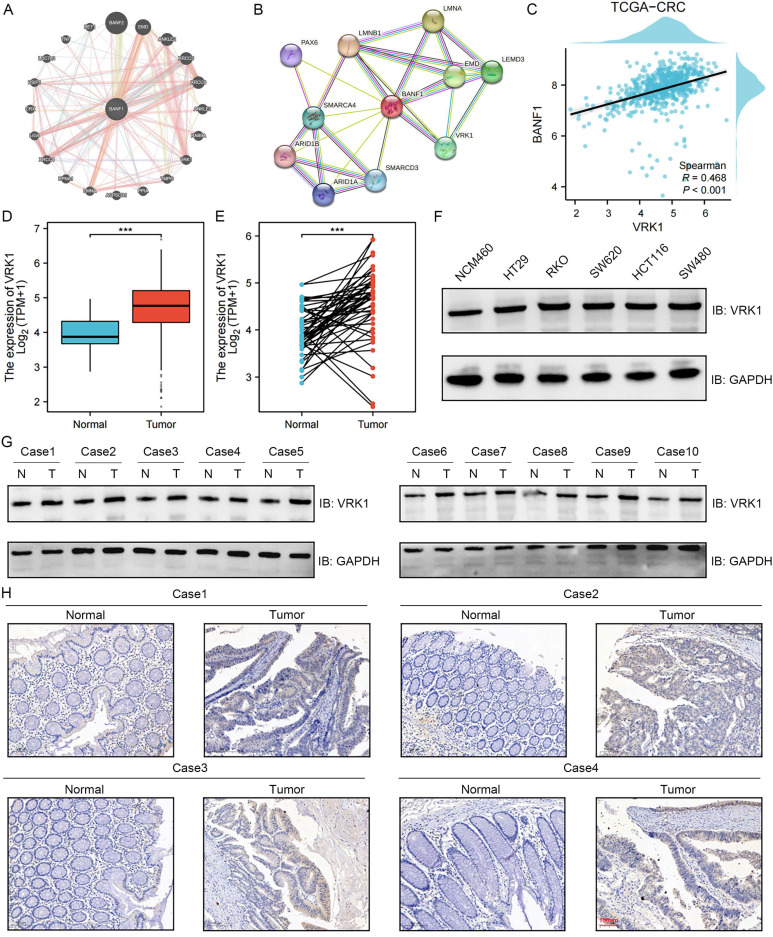
** Protein interaction prediction analysis suggested that VRK1 may interact with BANF1.** (A-B) GeneMANIA and STRING suggested a potential interaction between BANF1 and VRK1. (C) Co-expression analysis of VRK1 and BANF1 revealed a significant correlation between the transcript levels. (D-E) A significant increase in VRK1 mRNA expression in paired and unpaired CRC tissues compared to adjacent normal tissues. (F) VRK1 protein expression was higher in HT-29, RKO, SW-620, HCT-116, and SW-480 cells compared to NCM-460. (G) In 10 pairs of CRC samples, the majority of CRC tissues exhibited significantly higher VRK1 protein expression compared to adjacent non-cancerous tissues. (H) IHC results showed that the CRC tumor tissues exhibited a higher level of VRK1 protein expression. ***p < 0.001.

Protein interaction predictions were performed using two online tools, geneMANIA (Figure 7A) and STRING (Figure 7B), to construct protein interaction networks. Both analyses suggested a potential interaction between BANF1 and VRK1. Co-expression analysis revealed a significant correlation between the transcript levels of VRK1 and BANF1 (Figure 7C, *R* = 0.468, *p* < 0.001).

Given the lack of prior reports on VRK1's role in CRC, we examined VRK1 expression in both unpaired (Figure 7D) and paired (Figure 7E) CRC tumor and adjacent non-tumor tissues using data from the TCGA database. The study found a notable elevation in VRK1 mRNA levels in CRC tissues relative to adjacent normal tissues (*p* < 0.001). Validation through WB analysis in cell lines confirmed that VRK1 protein expression was higher in HT-29, RKO, SW-620, HCT-116, and SW-480 cells compared to NCM-460 cells (Figure 7F, Supplementary Figure S14A). Further validation with 10 paired CRC patient tissue samples showed that most CRC tissues had significantly elevated VRK1 protein levels compared to adjacent non-cancerous tissues (Figure 7G, Supplementary Figure S14B). IHC results further showed that CRC tumor tissues exhibited higher levels of VRK1 protein expression than adjacent normal tissues (Figure 7H).

### VRK1 regulates BANF1 protein expression and influences the proliferation, migration, and invasion of CRC cells

**Figure 8:**
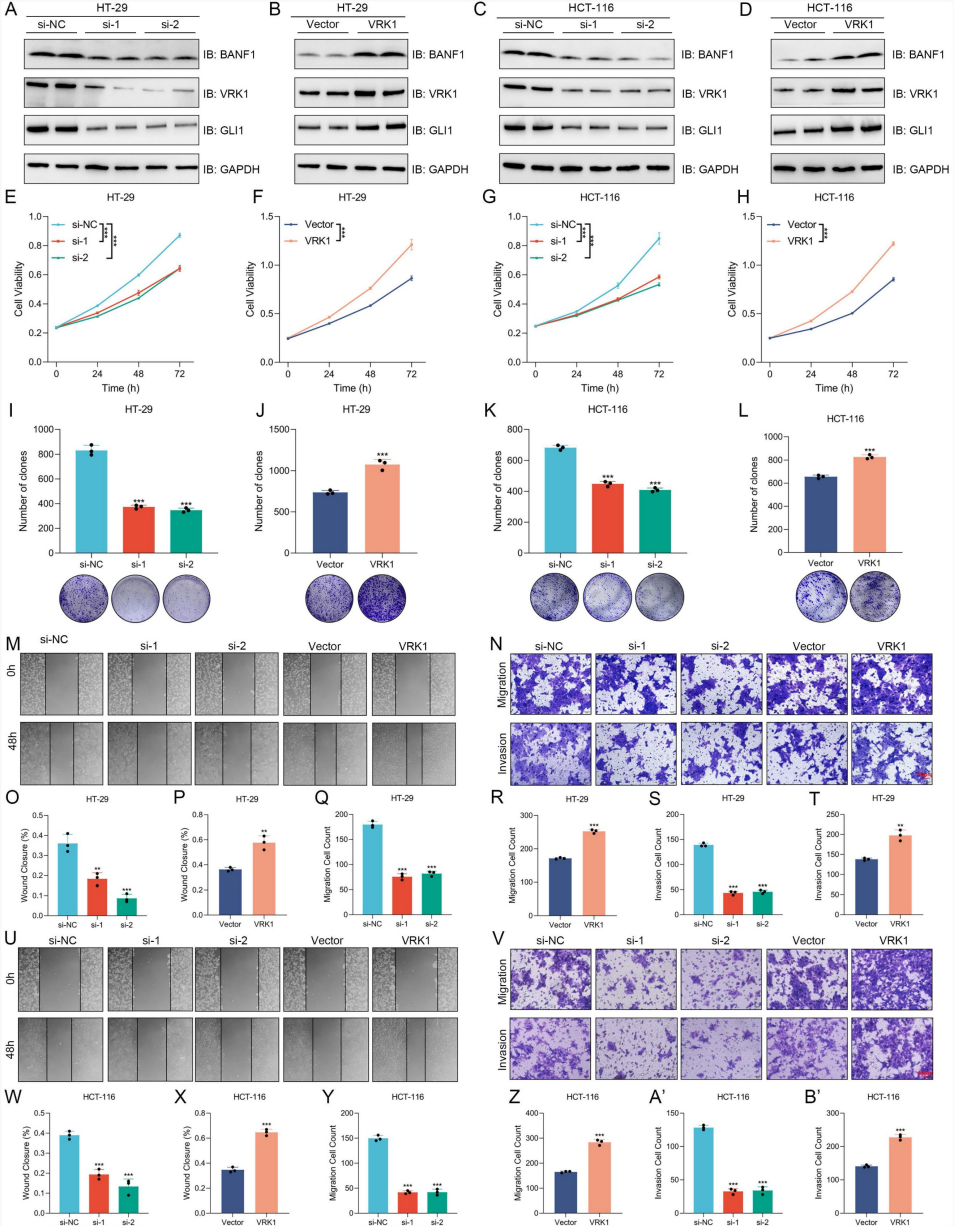
** VRK1 can regulate the protein expression of BANF1 and GLI1, as well as influence the proliferation, migration, and invasion of CRC cells.** (A-D) In VRK1-knockdown HT-29 and HCT-116 cells, the protein expression levels of BANF1 were reduced; conversely, in cells overexpressing VRK1, BANF1 protein levels were elevated. (E-H) Results of CCK-8 showed that VRK1 knockdown inhibited proliferation in both HT-29 and HCT-116 cell lines, whereas VRK1 overexpression markedly enhanced cell proliferation. (I-L) VRK1 knockdown led to a substantial reduction in colony formation capacity in both CRC cell lines, with a decrease in colony number. In contrast, VRK1 overexpression significantly promoted colony formation, resulting in a marked increase in colony number. (M, O-P) VRK1 knockdown significantly impaired the migration ability of HT-29 cells, whereas VRK1 overexpression markedly enhanced cell migration. (N, Q-T) VRK1 knockdown led to a significant reduction in the number of cells that migrated through the chamber, while VRK1 overexpression significantly increased the number of CRC cells that passed through the chamber. (U-B') Statistical analyses showed that VRK1 knockdown and overexpression respectively inhibited and promoted the migration and invasion abilities of CRC cells. **p < 0.01; ***p < 0.001.

In VRK1-knockdown HT-29 and HCT-116 cells, protein expression levels of BANF1 and GLI1 were significantly reduced (Figure 8A, C; Supplementary Figure S15A, C). Conversely, in cells overexpressing VRK1, BANF1 and GLI1 protein levels were elevated (Figure 8B, D; Supplementary Figure S15B, D). However, knockdown or overexpression of BANF1 did not significantly affect VRK1 protein expression in either cell line. VRK1 knockdown inhibited proliferation in both HT-29 (Figure 8E-F) and HCT-116 (Figure 8G-H) cell lines, whereas VRK1 overexpression markedly enhanced cell proliferation. Colony formation assays revealed that VRK1 knockdown significantly reduced colony formation capacity and decreased colony numbers in both CRC cell lines (Figure 8I, K). VRK1 overexpression significantly enhanced colony formation, resulting in a marked increase in colony numbers relative to the control group (Figure 8J, L). The cell scratch assay demonstrated that VRK1 knockdown significantly reduced HT-29 cell migration, whereas VRK1 overexpression notably increased it (Figure 8M, O-P). VRK1 knockdown significantly decreased cell migration through the chamber, while VRK1 overexpression enhanced the migration of CRC cells (Figure 8N, Q-T). Consistent results were observed in HCT-116 cells, where VRK1 knockdown inhibited and VRK1 overexpression promoted migration and invasion capabilities (Figure 8U-B').

### VRK1 and BANF1 may interact at the protein level

**Figure 9:**
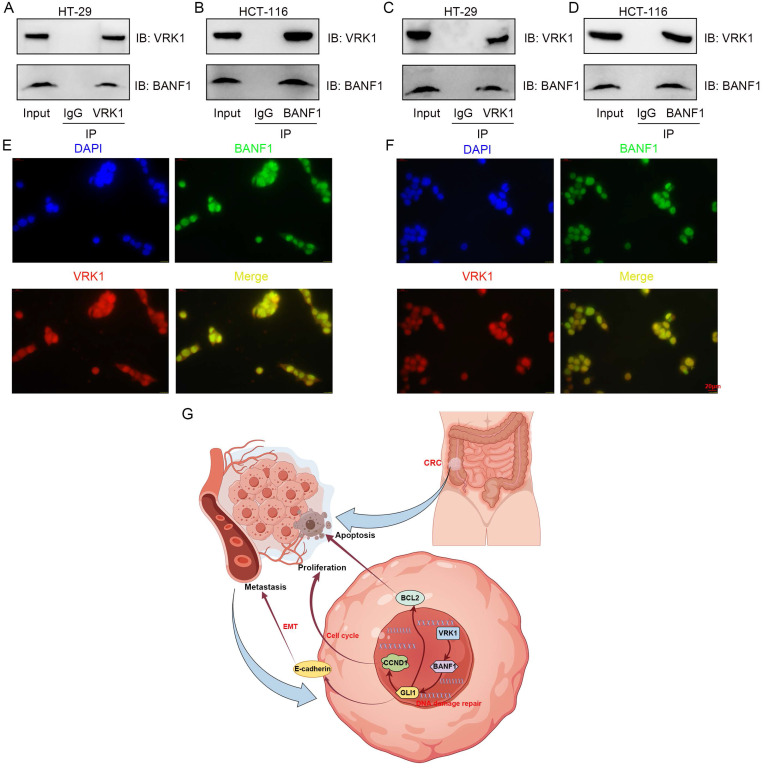
** VRK1 and BANF1 may exhibit potential interactions at the protein level.** (A-D) In both cell lines, after the addition of VRK1 or BANF1-specific antibodies, BANF1 or VRK1 proteins were detected in the precipitate, suggesting a potential interaction between VRK1 and BANF1 at the protein level. (E-F) Immunofluorescence colocalization further demonstrated that both VRK1 and BANF1 are expressed within the nucleus of HT-29 and HCT-116 cells. (G) A proposed mechanism that VRK1/BANF1/GLI1 axis regulates tumor development and progression of CRC, which was drawn using Figdraw online tool (https://www.figdraw.com/).

Co-immunoprecipitation and immunofluorescence colocalization assays were conducted in HT-29 and HCT-116 cell lines. Antibodies specific to VRK1 or BANF1 identified the corresponding proteins in the precipitate for both cell lines, indicating a potential interaction (Figure 9A-D). Immunofluorescence colocalization further confirmed nuclear expression of VRK1 and BANF1 in HT-29 and HCT-116 cells (Figure 9E-F).

### Diagnostic values and relevant biological functions of VRK1 and BANF1

Diagnostic values of VRK1 and BANF1 in various cancers were assessed using ROC analysis (Supplementary Figure S16A-B). GSEA revealed relevant biological functions of VRK1 in CRC (Supplementary Figure S17).

## Discussion

Recent studies have investigated the relationship between BANF1 and gastrointestinal tumors. Wang *et al.* 23 reported that the upregulation of BANF1 in tumor tissues is negatively correlated with immune cell infiltration. In immunocompetent mice, BANF1 deficiency in tumor cells significantly inhibited tumor growth and enhanced the response to immunotherapy in a colon cancer model. However, this effect was not observed in immunodeficient mice. A study 24 used IHC to evaluate BANF1 protein expression, revealing significantly elevated BANF1 levels in tumor tissues compared to adjacent non-tumor gastric mucosa. High BANF1 expression correlated with poor differentiation, increased invasion depth, lymph node metastasis, advanced tumor stage, and reduced overall and disease-free survival rates. In a prior study 25, machine learning algorithms were employed to identify potential diagnostic and therapeutic targets for gastric cancer. Database and experimental results revealed that BANF1 is localized in the nucleus of gastric cancer cells, with significantly elevated mRNA and protein expression levels in gastric cancer tissues and cell lines. Additionally, BANF1 knockdown significantly reduced the proliferation and migration capabilities of gastric cancer cells.

This study utilized public databases to analyze the transcriptomic expression levels of BANF1 across various malignancies. The results demonstrated a significant upregulation of BANF1 mRNA levels in CRC compared to adjacent non-tumor tissues. We confirmed these findings by evaluating BANF1 mRNA and protein expression in CRC cell lines and clinical tissue samples. Cox regression analyses and Kaplan-Meier survival curves indicated that elevated BANF1 expression correlates with reduced survival in CRC patients. To further aid prognosis prediction, a nomogram model was constructed, and its accuracy was validated using calibration curves. The model demonstrated strong predictive performance.

Additionally, a web-based calculator tool was developed to enable the convenient and accurate application of this prognostic model in clinical practice. Using a lentiviral system, we generated stable cell lines with upregulated or downregulated BANF1 expression in two CRC cell lines to perform phenotypic analyses. The experiments demonstrated that BANF1 knockdown significantly inhibited CRC cell growth both *in vitro* and *in vivo*, while its overexpression enhanced CRC proliferation. Furthermore, BANF1 knockdown and overexpression were shown to respectively reduce and increase CCND1 protein levels. Dysregulation of the cell cycle is a critical factor in the malignant proliferation of tumor cells, significantly influencing tumor growth 26-28. The cell cycle is meticulously controlled by key factors such as cyclin-dependent kinases, cyclins as positive regulators, and cyclin-dependent kinase inhibitors (CKIs) as negative regulators 29-31. Among these factors, CCND1 is frequently overexpressed in various tumors, and its imbalanced expression often leads to uncontrolled cell cycle progression, a key driver of tumorigenesis and progression 32. Previous studies 33-35 have highlighted the importance of CCND1 in CRC. For instance, MiR-519d enhances CRC cell sensitivity to 5-fluorouracil chemotherapy by downregulating CCND1, and Let-7i-3p suppresses CRC cell cycle progression, proliferation, migration, and invasion by downregulating CCND1. CDCA2 enhances CRC proliferation both *in vitro* and *in vivo* through activation of the AKT/CCND1 axis. Consistent with these findings, we observed that BANF1 knockdown suppressed CRC proliferation while downregulating CCND1.

Our findings indicate that BANF1 affects CRC cell migration and invasion *in vitro* and *in vivo*, and modulates EMT-related proteins associated with CRC metastasis. For instance, GOLM1 promotes CRC progression and metastasis via the AKT/GSK3β/EMT axis 36, while ADAMDEC1 enhances metastasis by inducing EMT and amplifying Wnt signaling 37. Flow cytometry showed that BANF1 knockdown induces apoptosis in CRC cells, inhibiting progression, with changes in apoptosis-related proteins BCL2 and cleaved-caspase-3. Similarly, a previous study demonstrated that CTSG regulates apoptosis in CRC by modulating BCL2 expression, thereby influencing progression 38. Xu *et al.* 39 reported that patients with CRC undergoing surgery and radiotherapy benefited more than those undergoing surgery alone. Using colony formation assays, we found that BANF1 knockdown significantly increased CRC sensitivity to radiotherapy and reduced DNA repair proteins DNA-PKcs, KU70, KU80, and RAD51. Previous research indicated that ITGB5 decreases pancreatic cancer sensitivity to radiotherapy by promoting DNA damage repair and activating the MEK/ERK pathway. ITGB5 knockdown significantly reduced DNA-PKcs, KU70, KU80, and RAD51 expression 40, consistent with our findings.

We performed RNA-seq and bioinformatics analysis on HCT-116 cells with BANF1 knockdown and control HCT-116 cells, followed by WB experiments to validate the impact of BANF1 knockdown and overexpression on the expression of the Hedgehog pathway protein GLI1. Recent research has highlighted GLI1 as a key regulator in the development and progression of multiple cancers. For instance, RCC2 promotes prostate cancer cell proliferation and migration through the Hedgehog/GLI1 signaling pathway 41, while MT1M regulates gastric cancer progression and stemness by modulating GLI1 42. In cervical cancer, PRKCI mediates radiosensitivity via the Hedgehog/GLI1 pathway 43, and Garcinone C inhibits the tumorigenic and invasive potential of CRC stem-like cells by targeting the Hedgehog/GLI1 signaling pathway 44. To investigate whether BANF1 mediates CRC proliferation and migration through the regulation of GLI1 expression, we performed rescue experiments by introducing GLI1 overexpression plasmids and siRNA into BANF1 knockdown and overexpressing stable cell lines. The results indicated that the effects of BANF1 on CRC cell proliferation and migration could be reversed by GLI1. Additionally, the expression levels of GLI1, CCND1, E-cadherin, and BCL2 proteins were correspondingly altered. A previous study 45 demonstrated that GLI1 regulates CCND1, BCL2, and EMT-related proteins. These findings suggest that BANF1 influences CRC cell proliferation, migration, and apoptosis by regulating GLI1 expression and subsequently modulating the expression of proliferation, EMT, and apoptosis-related proteins.

To further explore proteins interacting with BANF1, we used two online tools for protein interaction prediction, which suggested that VRK1 might interact with BANF1. Data from the Oncomine database indicated that VRK1 is overexpressed in nearly all tumor types 46. Microarray data analysis has confirmed this overexpression in several cancers, such as breast 47,48, lung 49,50, head and neck squamous cell carcinoma 51, liver 52, glioma 53, multiple myeloma 54,55, and esophageal cancer 56. VRK1 overexpression in the human kinome is linked to poor prognosis and heightened proliferation in aggressive breast cancer, especially in estrogen receptor-positive (ER+) tumors 47,57,58. VRK1 depletion affects nuclear envelope morphology and causes BANF1 retention on mitotic chromosomes 59. Additionally, VRK1 can mediate the phosphorylation of BANF1 60. A recent study 61 suggested that VRK1 may act as an upstream regulator of BANF1, influencing esophageal cancer proliferation and migration phenotypes 62. Analysis of the TCGA database demonstrated a significant positive correlation between VRK1 and BANF1 mRNA expression levels in CRC tissues, with VRK1 expression notably elevated. Subsequent WB and IHC experiments confirmed this observation. We explored the functional relationship between VRK1 and BANF1 using siRNA and overexpression plasmids for transient transfection. Knocking down VRK1 in HT-29 and HCT-116 colorectal cancer cell lines led to reduced BANF1 protein levels, which was associated with inhibited cell proliferation and migration. Conversely, VRK1 overexpression increased BANF1 protein levels, enhancing CRC cell proliferation and migration. Co-immunoprecipitation and immunofluorescence co-localization experiments further suggested that VRK1 and BANF1 may interact at the protein level. A proposed mechanism illustrating the regulation of CRC tumor development and progression by the VRK1/BANF1/GLI1 axis is shown in Figure 9G.

Nonetheless, this research possesses specific limitations. Firstly, the number of clinical samples included was relatively small, which may limit the generalizability of our findings. Larger sample sizes will be necessary in future studies to further validate our conclusions. Secondly, the precise mechanisms by which BANF1 regulates GLI1 expression in CRC, and how VRK1 mediates BANF1 expression, remain incompletely understood. Additional studies are needed to clarify the specific molecular mechanisms of these regulatory interactions.

## Supplementary Material

Supplementary figures and tables.

## Abbreviations

BANF1: Barrier to Autointegration Factor 1
CRC: colorectal cancer
WB: Western blotting
IHC: immunohistochemistry
RNA-seq: RNA sequencing
PD-1: programmed death receptor-1
DNA-PK: DNA-dependent protein kinase
BSA: bovine serum albumin
PBS: phosphate buffered saline
ABC: avidin-biotin complex
FBS: fetal bovine serum
KM: Kaplan-Meier
CEA: carcinoembryonic antigen
COAD: colon adenocarcinoma
READ: rectum adenocarcinoma
SD: standard deviation
GSEA: Gene Set Enrichment Analysis
EMT: epithelial-mesenchymal transition
GO: Gene Ontology
KEGG: Kyoto Encyclopedia of Genes and Genomes
DO: Disease Ontology
CDKs: cyclin-dependent kinases
CKIs: cyclin-dependent kinase inhibitors
