# Supplementary material for: VRK1/BANF1/GLI1 Axis Regulates Tumor Development and Progression of Colorectal Cancer
Source: Int J Biol Sci. 2025 Apr 28;21(7):3144–63. doi: 10.7150/ijbs.107279 (PMC12080402; doi:10.7150/ijbs.107279)
Supplement: Supplementary file 1 — Supplementary figures and tables. [file ijbsv21p3144s1.pdf]

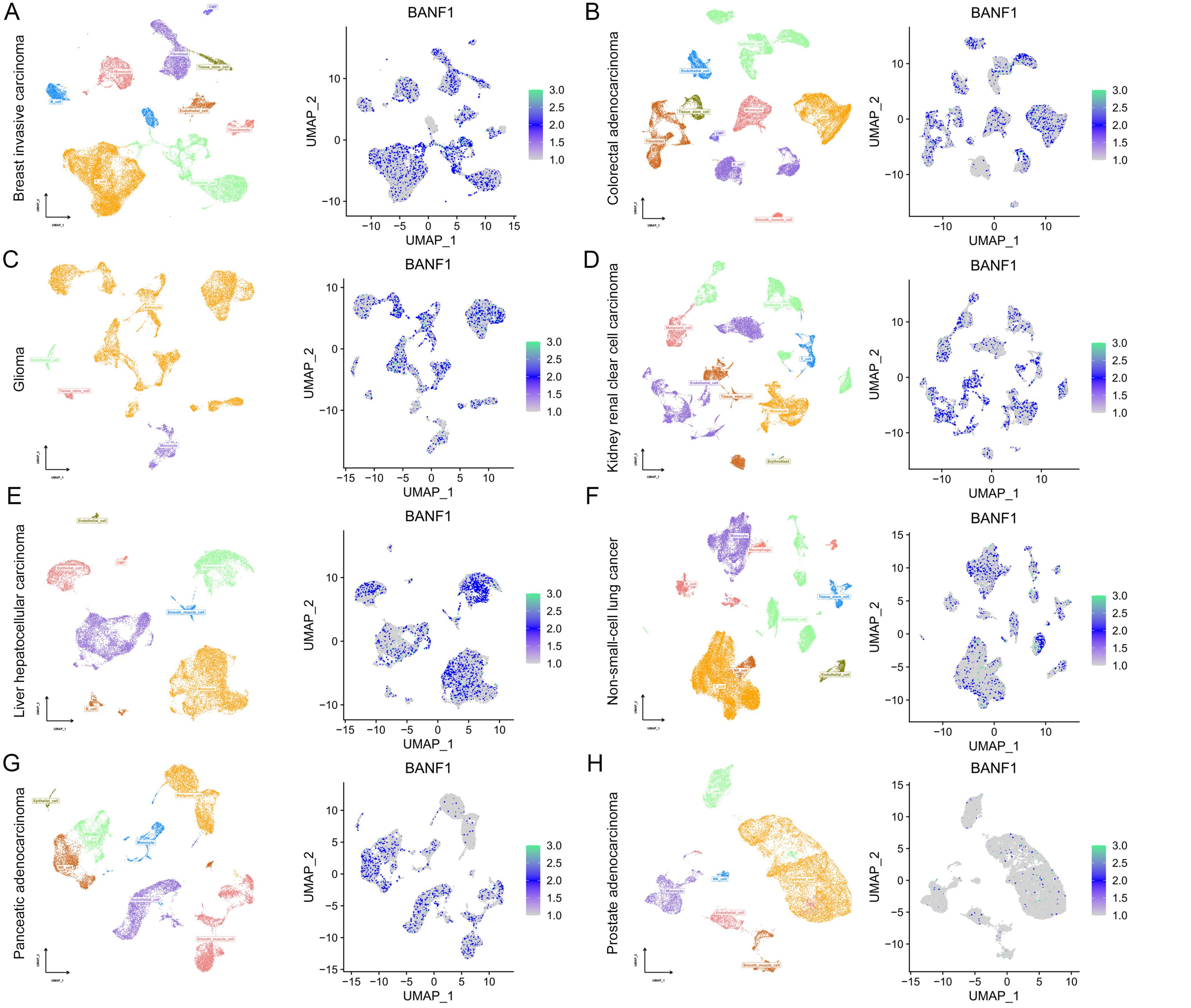

Supplementary Figure S1: BANF1 is extensively expressed across a range of cell types, including epithelial cells, tumor cells, and immune cells, in various malignant tumors such as invasive breast cancer (A), colorectal adenocarcinoma (B), glioma (C), clear cell renal carcinoma (D), hepatocellular carcinoma (E), non-small cell lung cancer (F), pancreatic cancer (G), and prostate cancer (H).

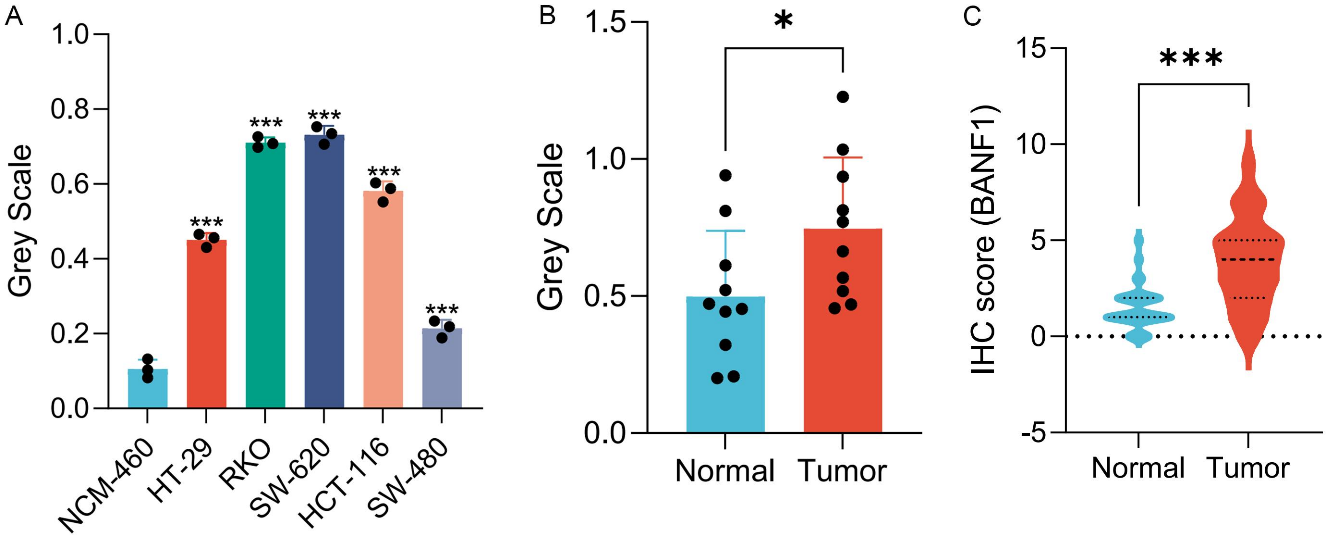

Supplementary Figure S2: The quantitative analysis of western blotting data for BANF1 in CRC cell lines (A) and tissues (B), along with IHC staining results from 68 pairs of CRC and adjacent normal tissues (C), is presented. *$p < 0.05$; ***$p < 0.001$.

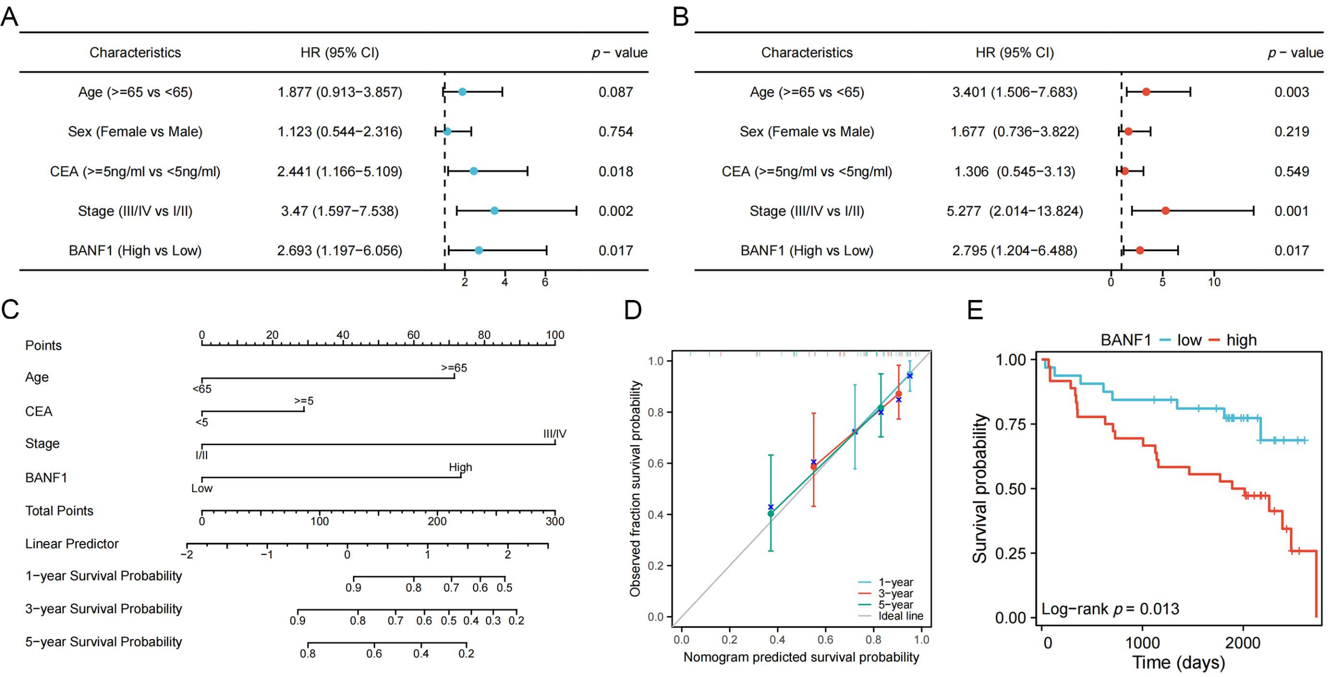

Supplementary Figure S3: The findings from the univariate (A) and multivariate (B) Cox regression analyses indicate that BANF1 exhibits strong predictive capabilities for overall survival. By incorporating BANF1 expression along with other key clinical variables such as age, preoperative CEA levels, and AJCC staging, a nomogram model was constructed (C). The accuracy of this nomogram was assessed through a calibration plot (D). Additionally, Kaplan-Meier survival analyses revealed that colorectal cancer (CRC) patients with low BANF1 expression had a significantly better prognosis, characterized by longer survival times, compared to those with high BANF1 expression (E).

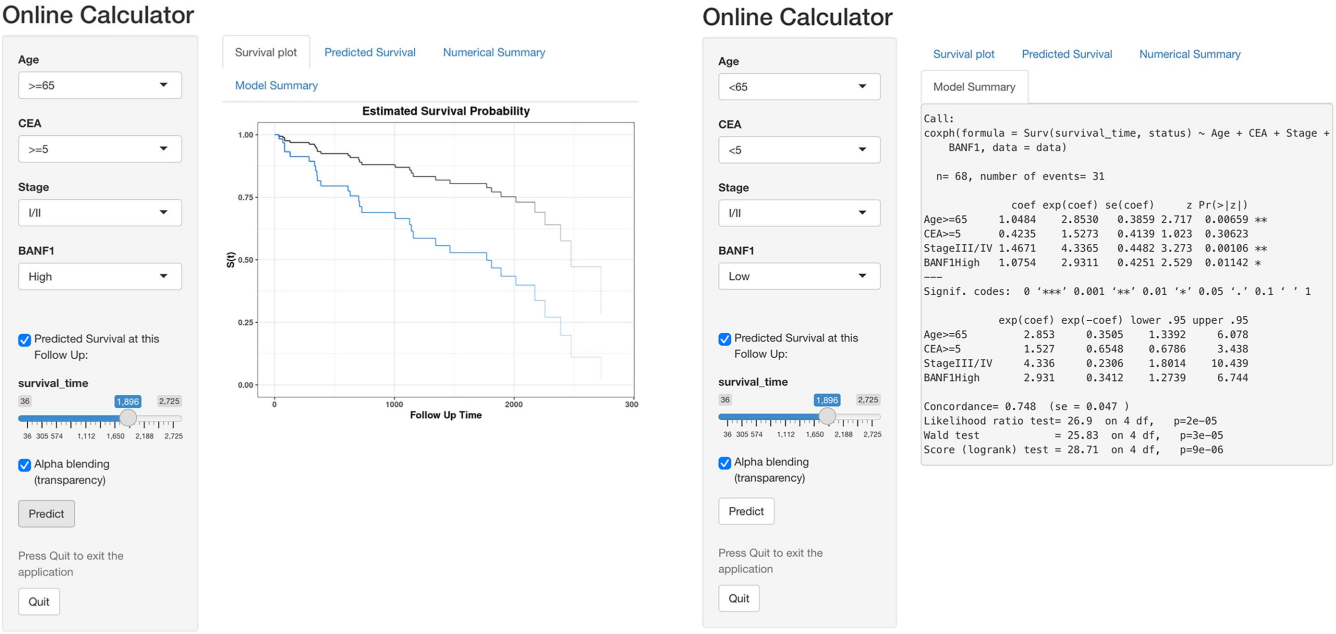

Supplementary Figure S4: To enhance the clinical applicability of this model, we have developed an online web-based calculator (accessible at https://doctorwang.shinyapps.io/BANF1/), designed to offer valuable insights for clinical practice and inform treatment decision-making.

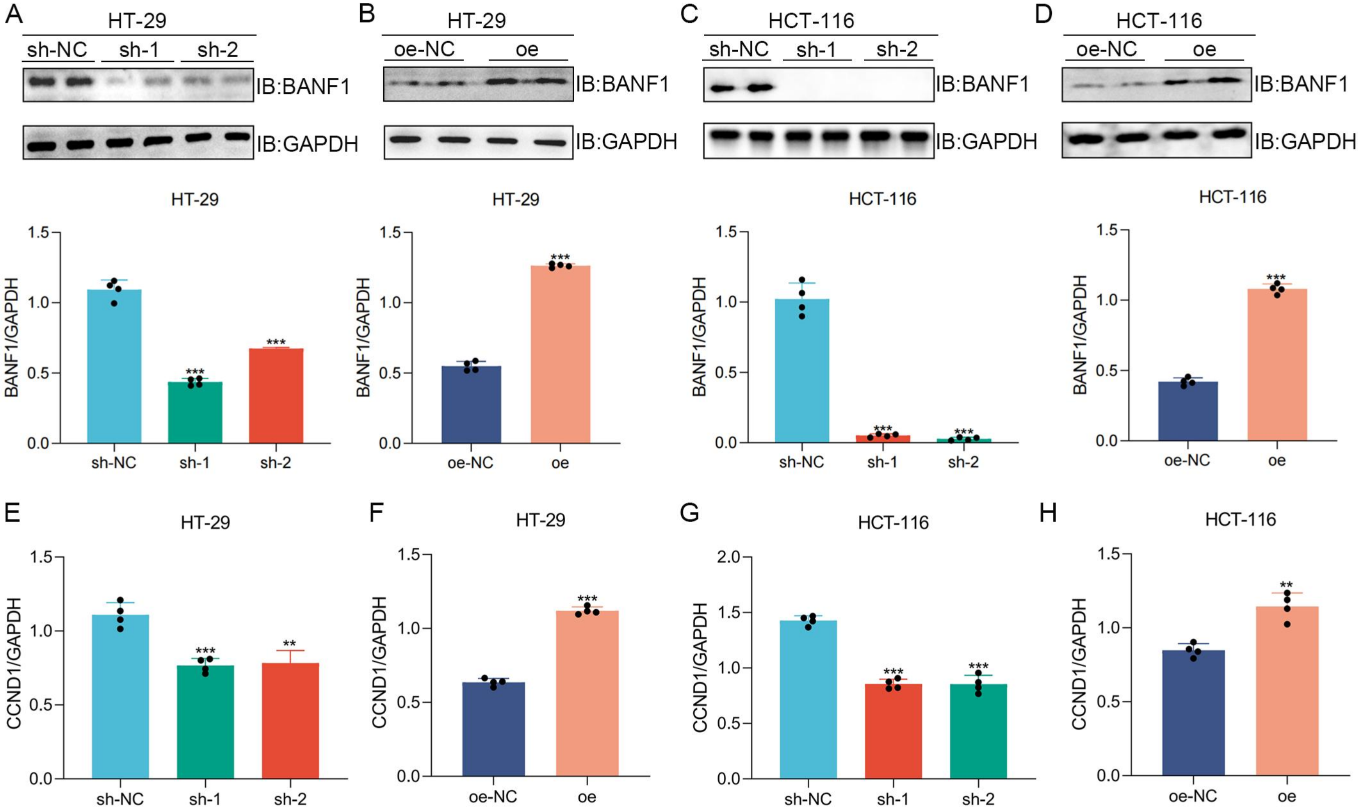

Supplementary Figure S5: (A-D) The efficacy of BANF1 knockdown and overexpression was further corroborated using Western blot analysis. (E-H) A quantitative analysis of the western blotting results for CCND1 was also conducted. $**p < 0.01$; $***p < 0.001$.

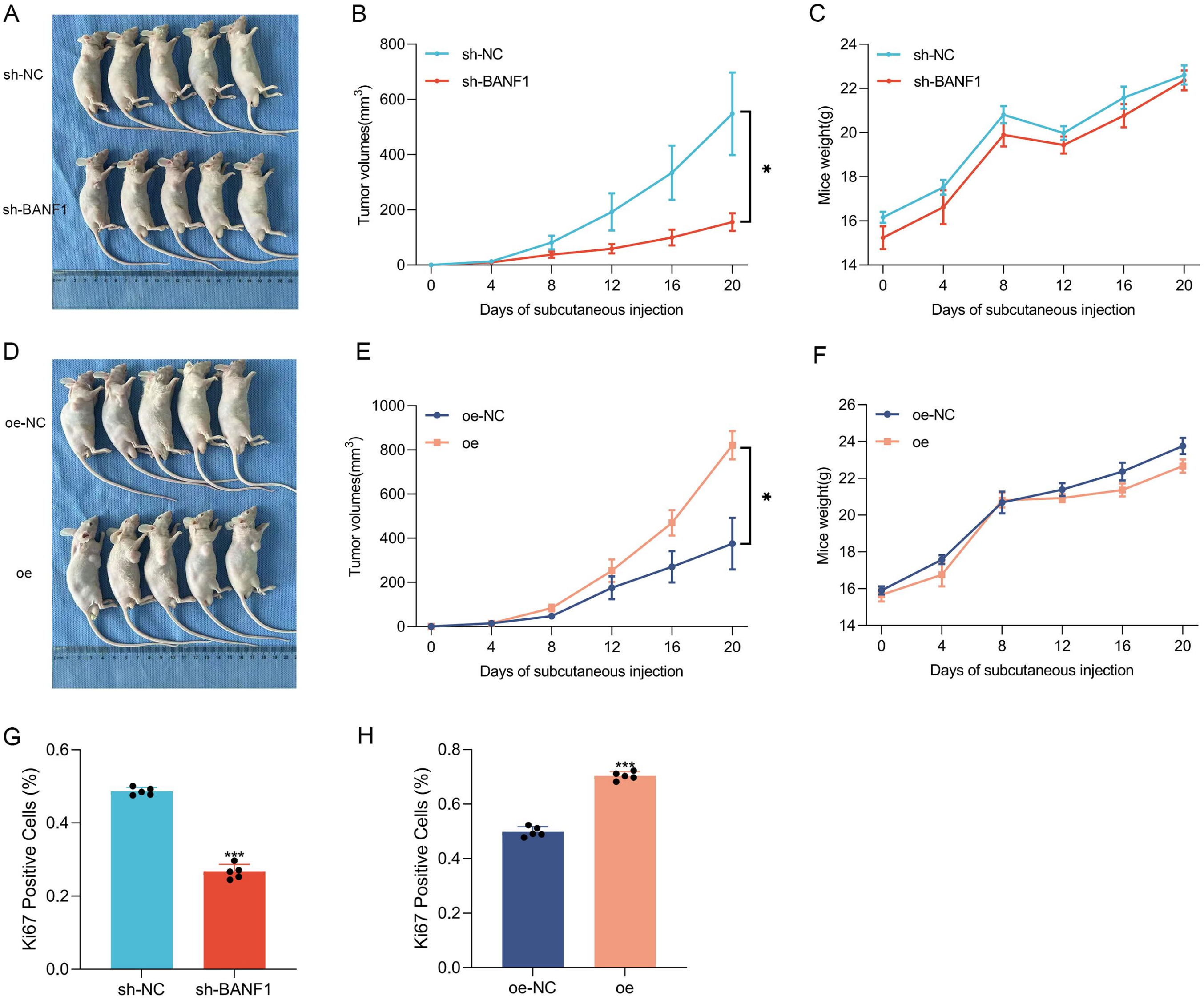

Supplementary Figure S6: Mice were injected with control and BANF1 knockdown cells (A), and measurements of tumor volume (B) and body weight (C) were recorded every four days. Similarly, mice injected with control and BANF1 overexpression cells (D) had their tumor volumes (E) and body weights (F) documented. The tumors from the BANF1 knockdown group exhibited lower Ki67 staining intensity, the oe group showed higher Ki67 staining intensity compared to the oe-NC group. ***$p < 0.001$.

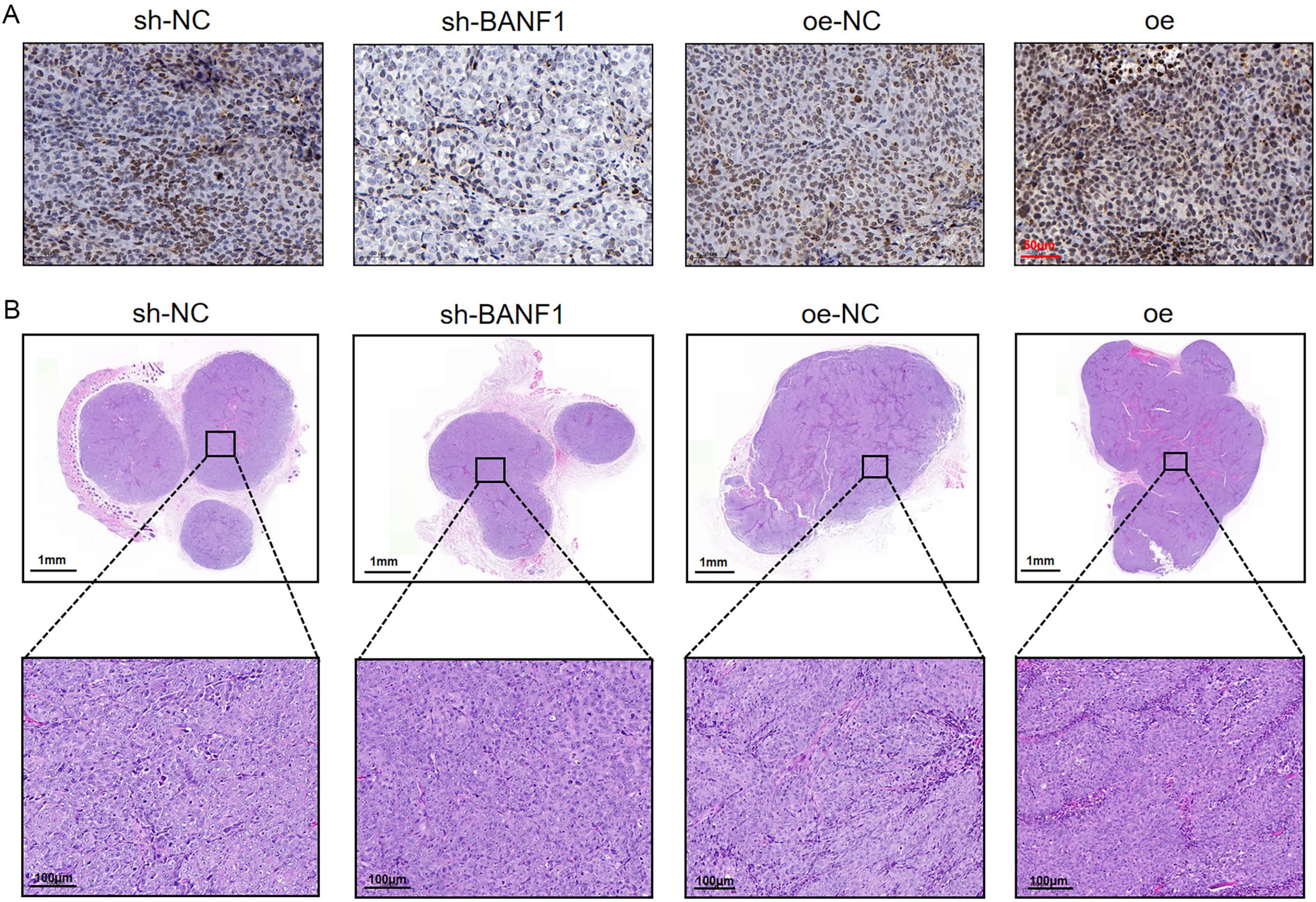

Supplementary Figure S7: (A) IHC staining analysis of BANF1 in subcutaneous tumor models utilizing nude mice. (B) Representative HE staining images illustrating BANF1 expression in subcutaneous tumor models.

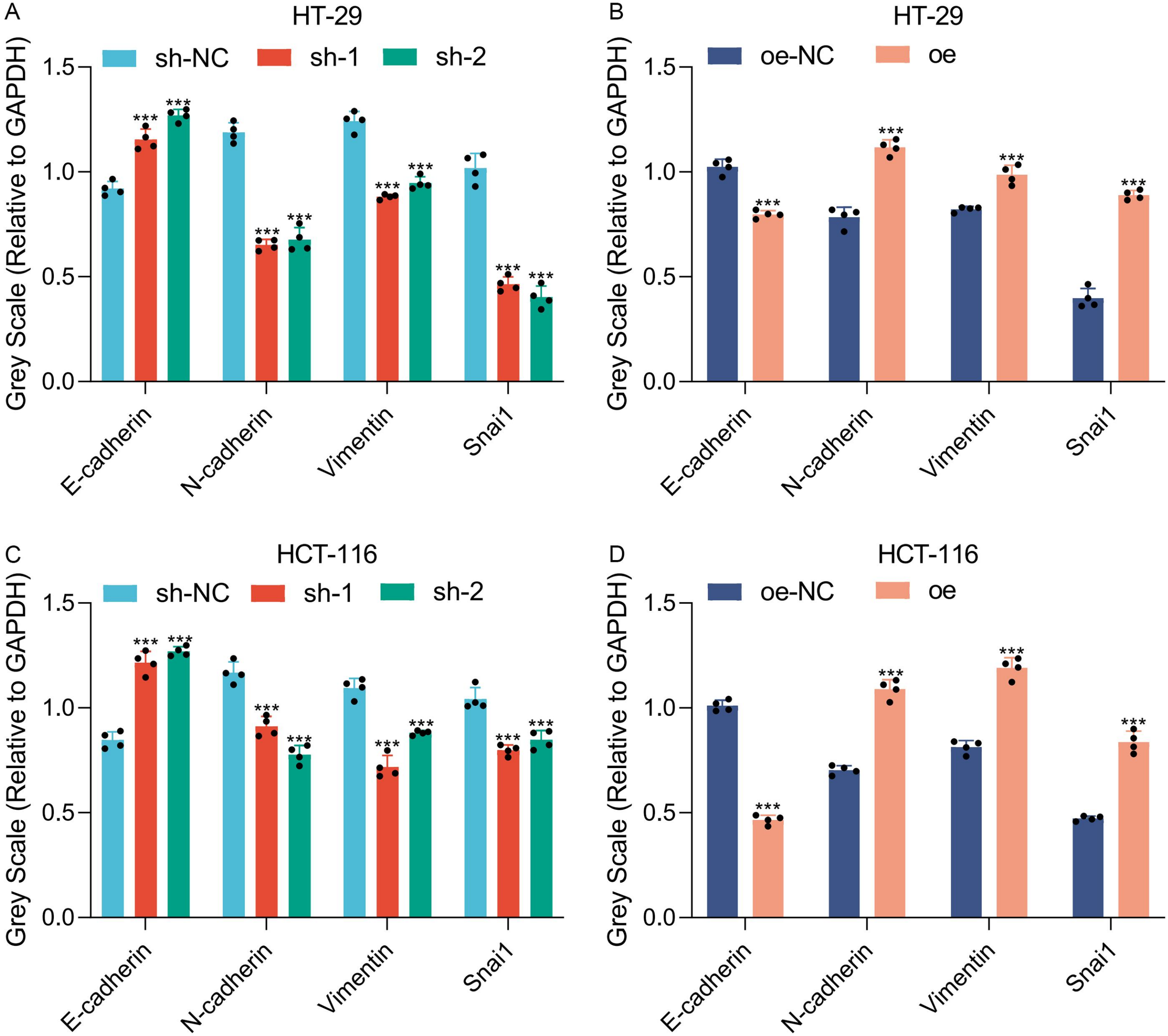

Supplementary Figure S8: (A-D) A quantitative assessment of the Western blot results for epithelial-mesenchymal transition (EMT)-related markers in cell lines with BANF1 knockdown and overexpression. ***$p < 0.001$.

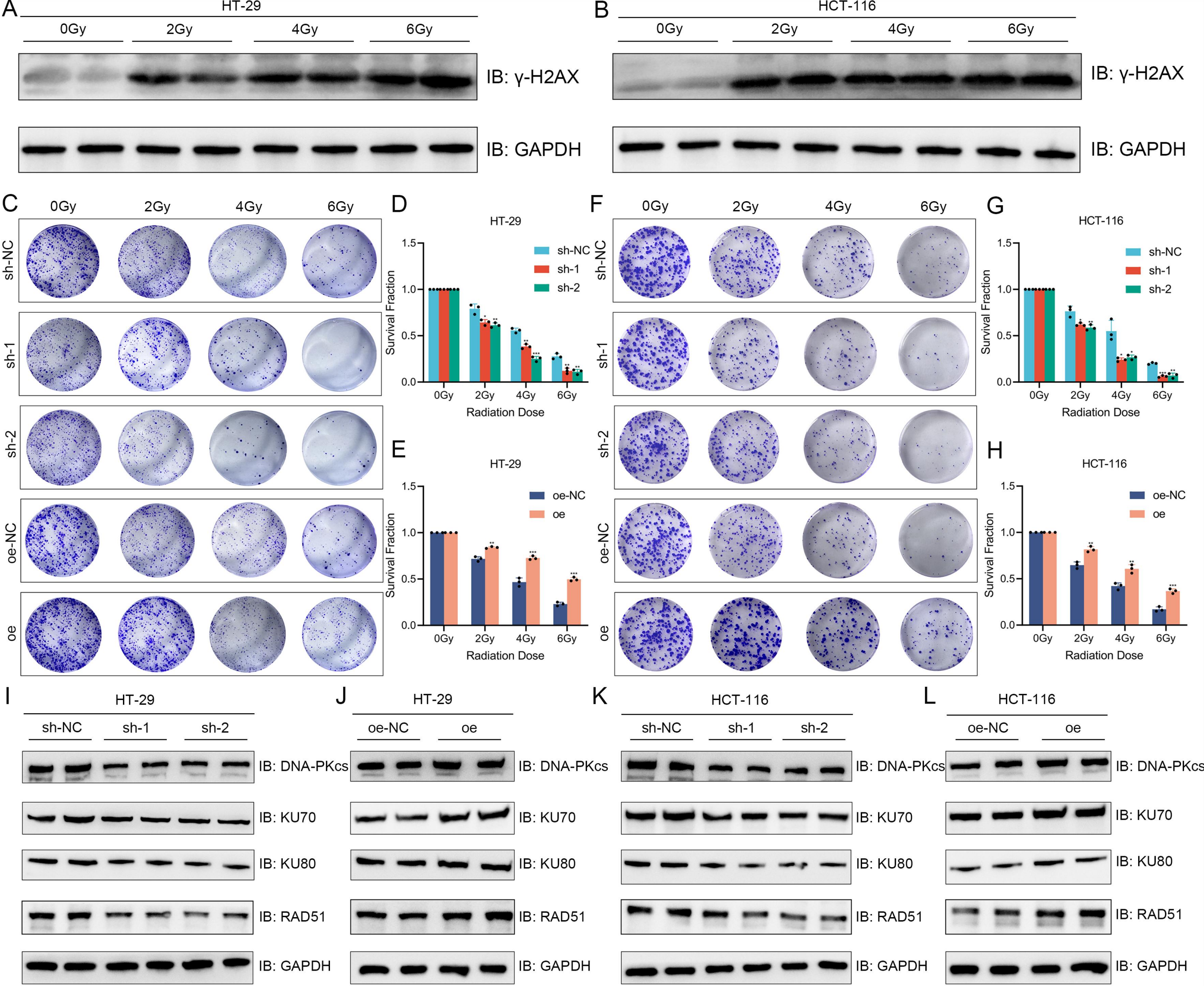

Supplementary Figure S9: (A-B) As radiation dose increases, γ-H2AX protein levels rise in HT-29 and HCT-116 cellsAs radiation dose increases, γ-H2AX protein levels rise in HT-29 and HCT-116 cells. (C-E) In HT-29, BANF1 knockdown reduces colony survival at 2 Gy, 4 Gy, and 6 Gy, while overexpression increases survival. (F-H) In HCT116, BANF1 knockdown enhances radiotherapy sensitivity, whereas overexpression decreases it. (I-L) Both cell lines show reduced DNA-PKcs, KU70, KU80, and RAD51 levels in sh-1 and sh-2 groups compared to sh-NC, while overexpression elevates these levels compared to oe-NC. *$p < 0.05$; **$p < 0.01$; ***$p < 0.001$.

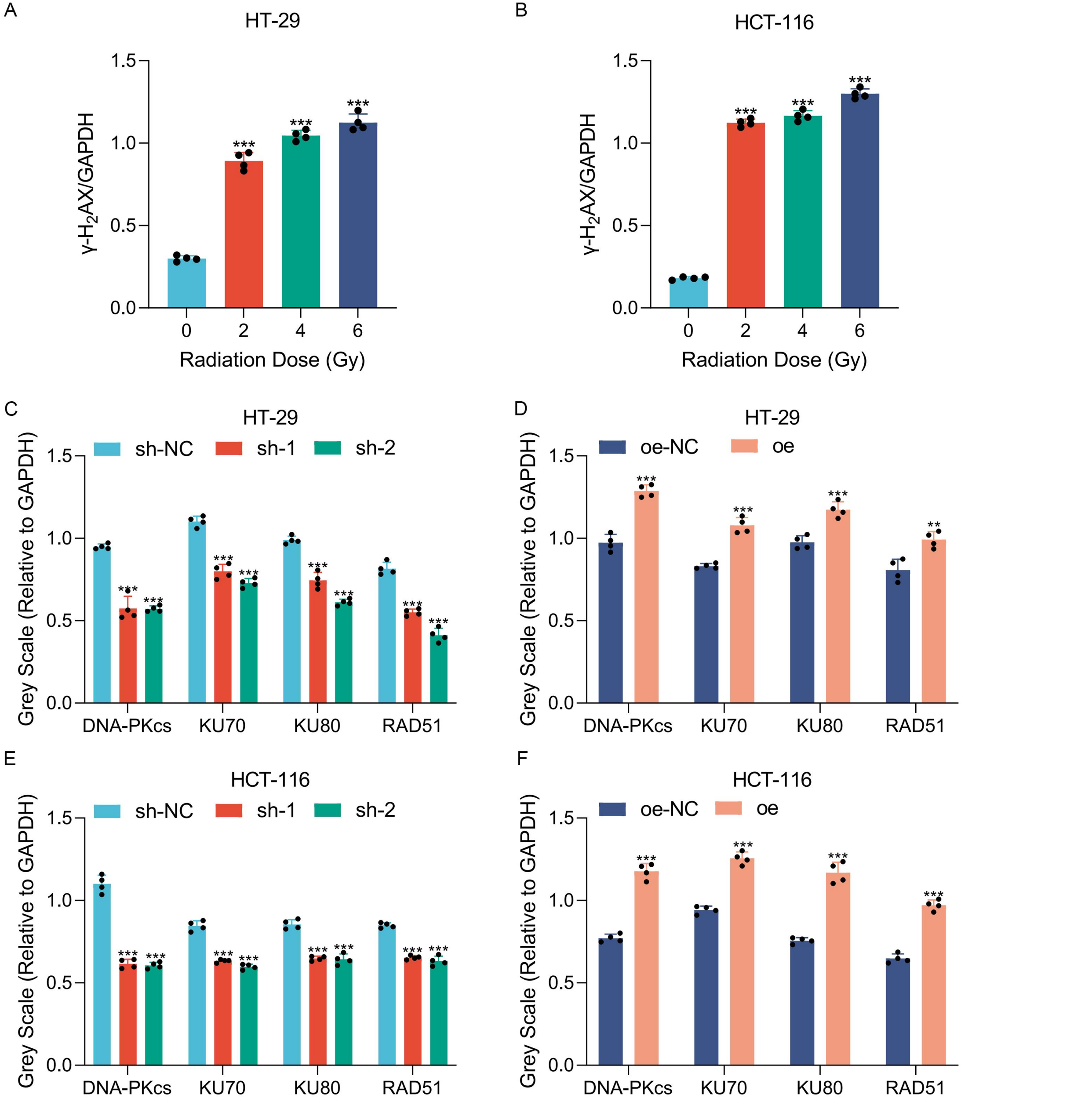

Supplementary Figure S10: The gray scale analysis of γ-H2AX in HT-29 (A) and HCT-116 (B) cell lines following radiation treatment is presented. (C-F) The quantitative analysis of western blotting results pertaining to DNA damage repair-related markers in cell lines with BANF1 knockdown and overexpression were shown. $**p < 0.01$; $***p < 0.001$.

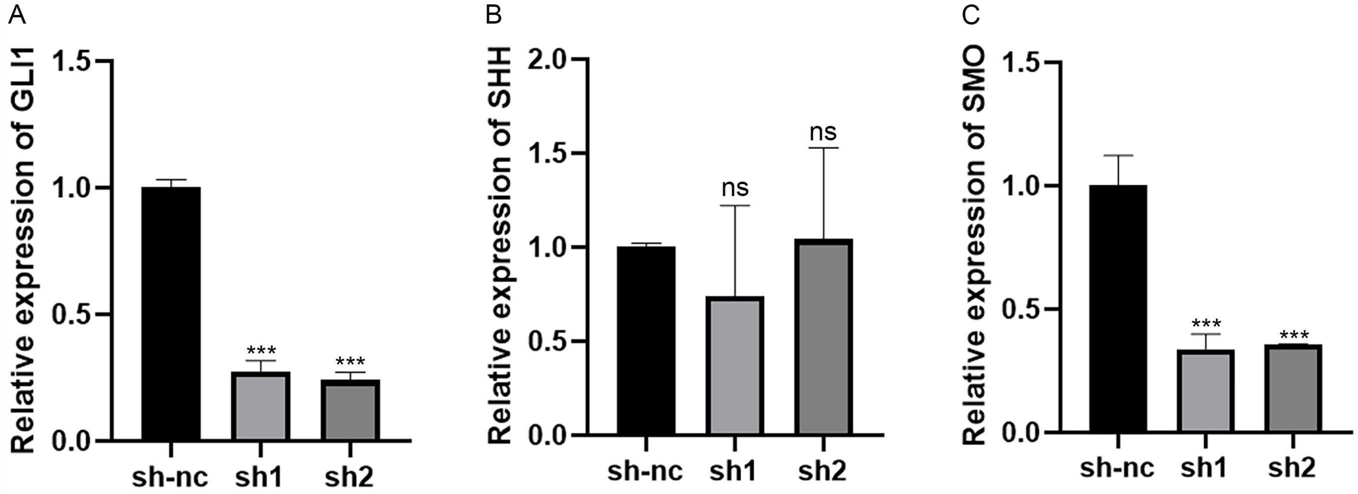

Supplementary Figure S11: qRT-PCR analysis was conducted to assess the mRNA expression levels of markers associated with the Hedgehog signaling pathway in both control and BANF1 knockdown HCT-116 cell lines. The markers analyzed included GLI1 (A), SHH (B), and SMO (C). ns: no significance; $***p < 0.001$.

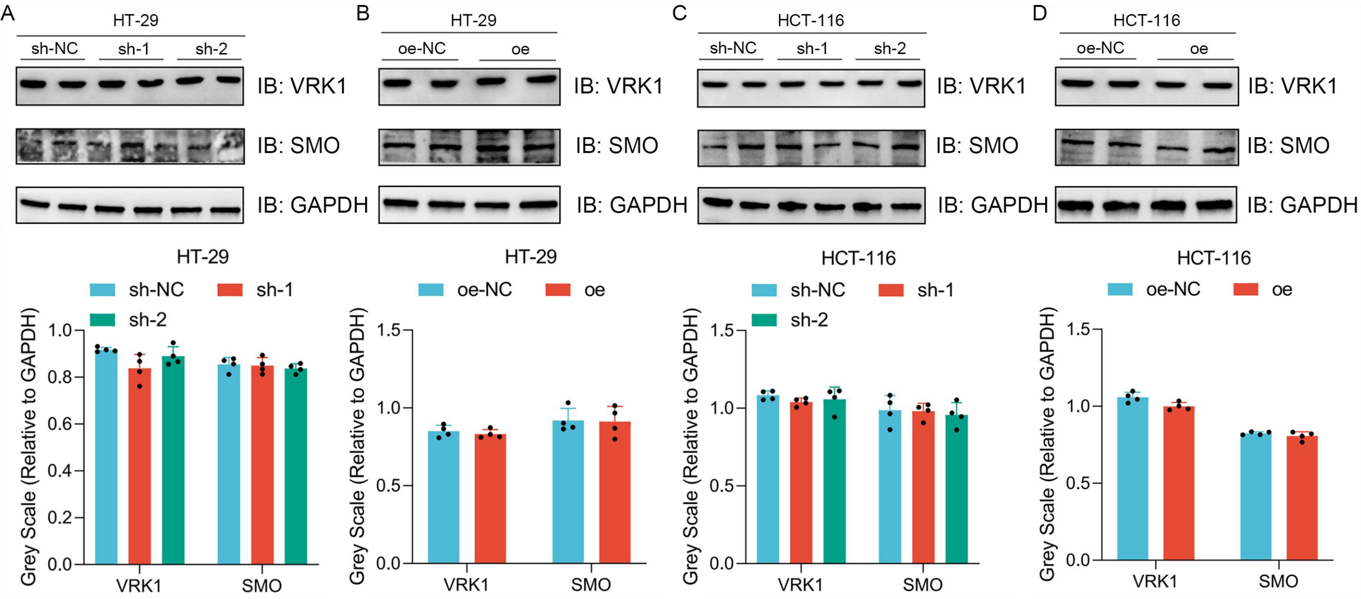

Supplementary Figure S12: (A-D) The modulation of BANF1 expression, either through knockdown or overexpression, did not affect the protein levels of VRK1 and SMO.

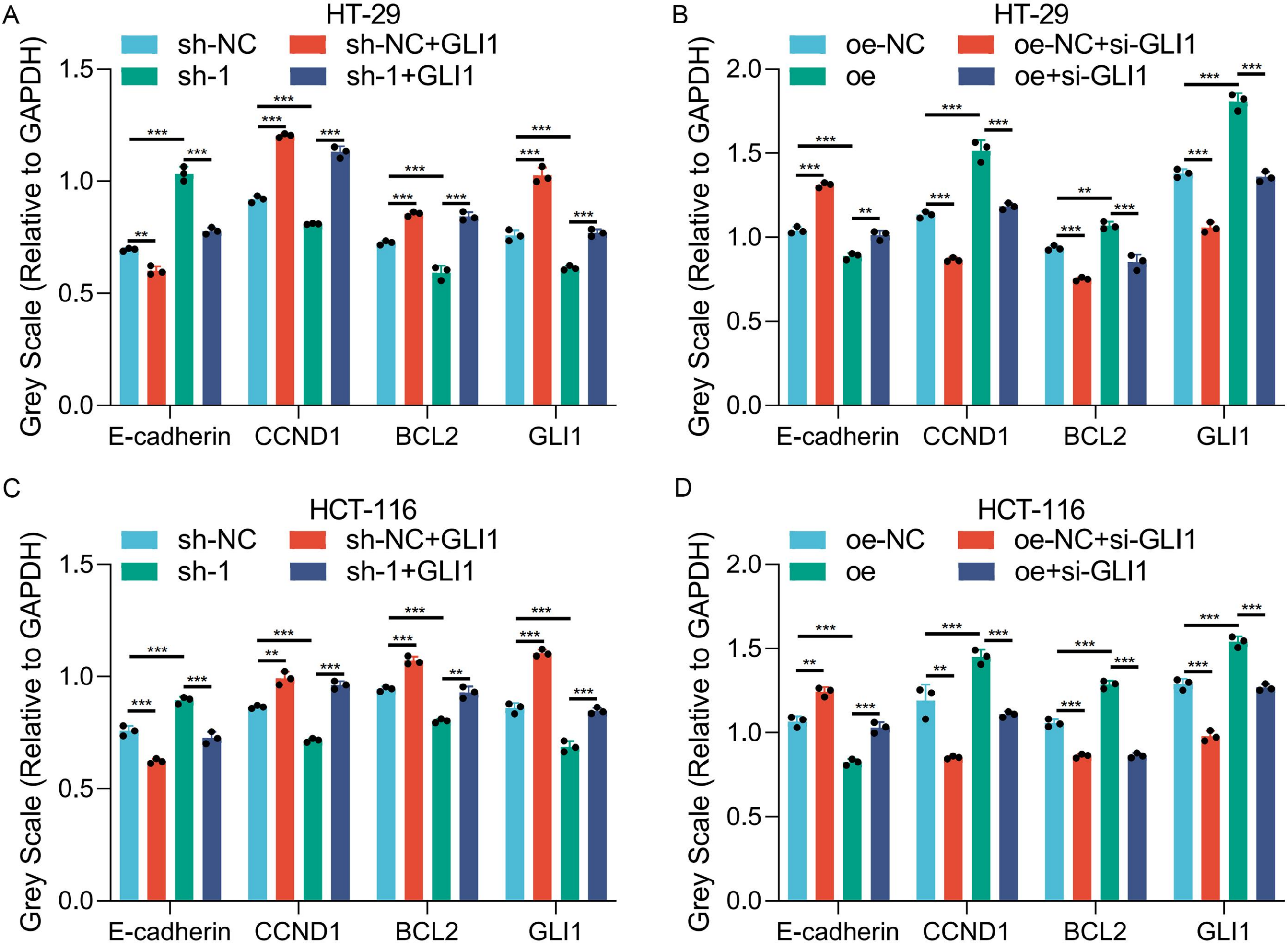

Supplementary Figure S13: (A-D) Quantitative analysis of the results obtained from western blotting in the context of rescue experiments. $**p < 0.01$; $***p < 0.001$.

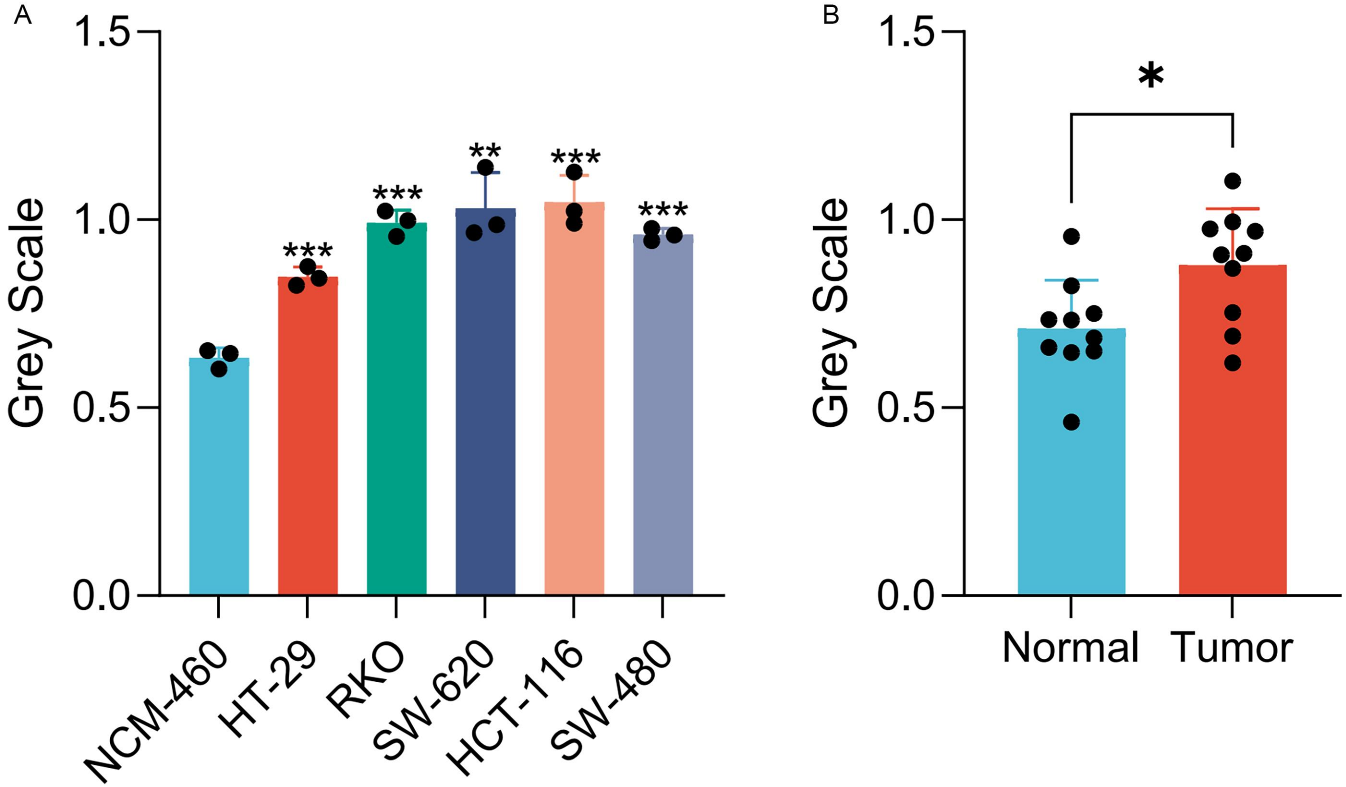

Supplementary Figure S14: Quantitative analysis of Western blot data for VRK1 expression in CRC cell lines (A) and tissue samples (B). *$p < 0.05$; **$p < 0.01$; ***$p < 0.001$.

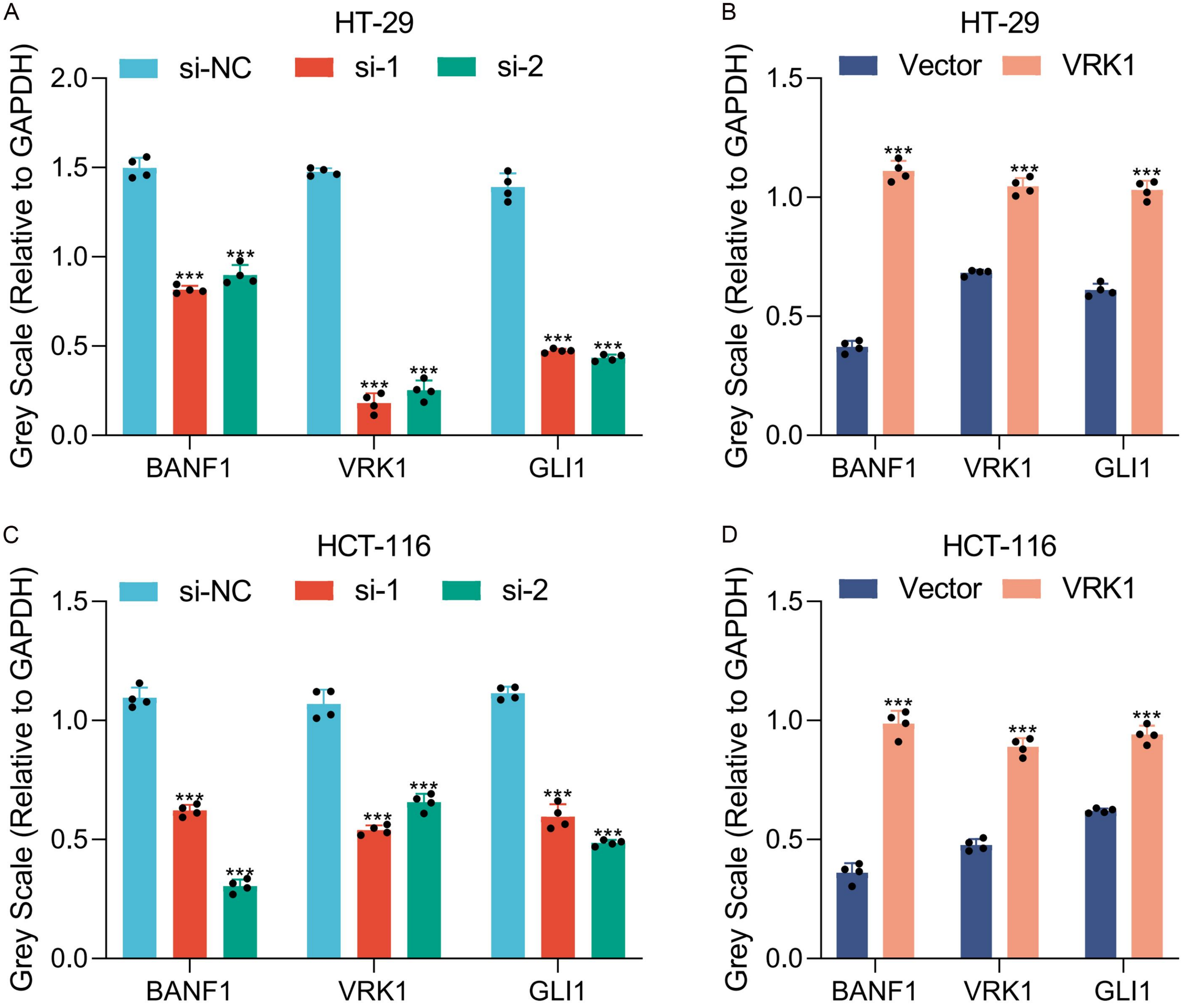

Supplementary Figure S15: (A-D) Quantitative analysis of western blot results for BANF1, VRK1, and GLI1 following VRK1 knockdown and overexpression in HT-29 and HCT-116 cell lines. ***$p < 0.001$.

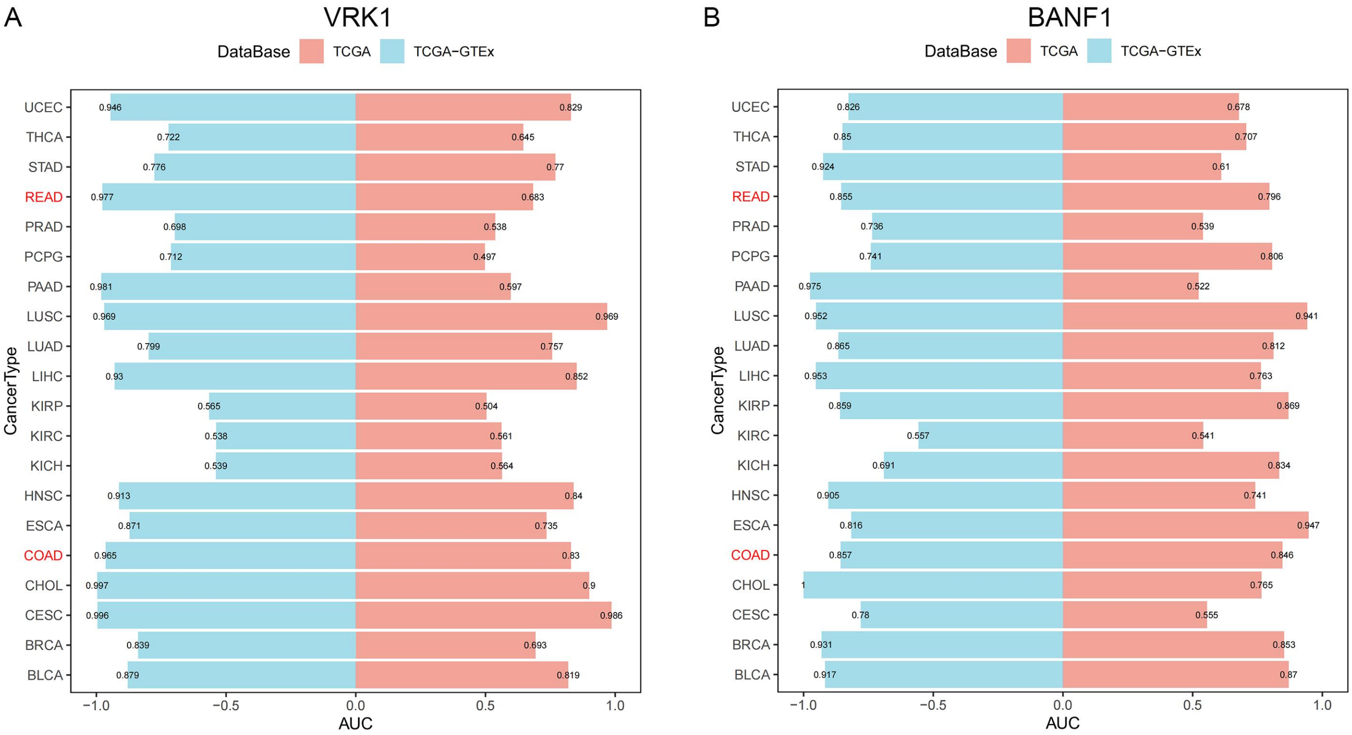

Supplementary Figure S16: The AUC values for VRK1 (A) and BANF1 (B) in both normal and tumor tissues were analyzed using the TCGA and GTEx datasets.

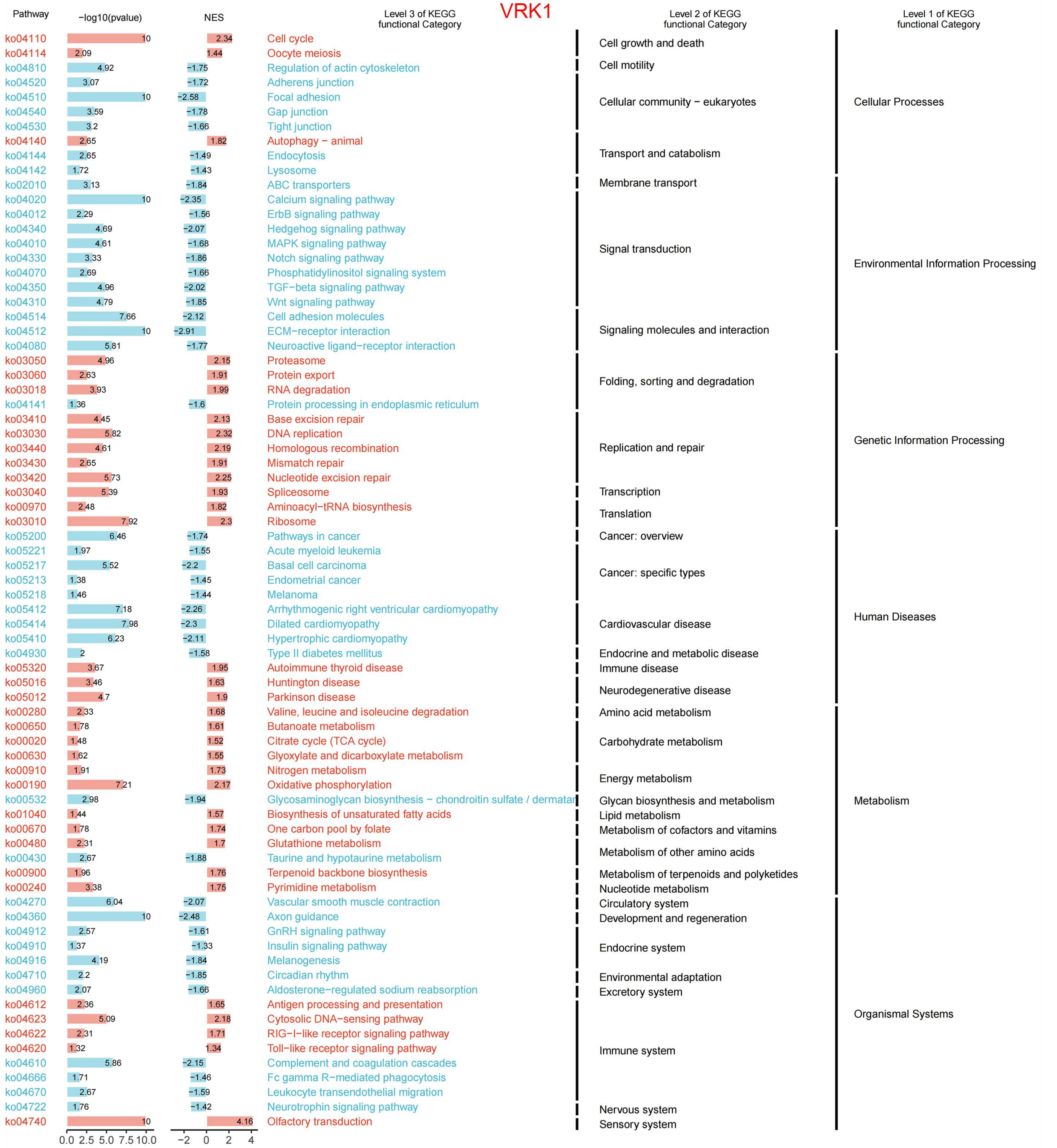

Supplementary Figure S17: GSEA was conducted to assess the biological functions and associated pathways of VRK1.

Supplementary Table S1: The primer sequences for PCR amplification.

Gene	Forward primer (5' to 3')	Reverse primer (5' to 3')
BANF1	TGGCTGAAAGACACTTGTGG	CACTCTCGAAGGCATCCGAAG
GAPDH	GGGAAGGTGAAGGTCGGAGT	GGGGTCATTGATGGCAACA

Supplementary Table S2: Antibodies used in this study.

Anti-BANF1 antibody (Zhengneng Biological, China), used for immunohistochemistry;

Anti-BANF1 antibody (Santa Cruz Biotechnology, USA), used for western blotting;

Anti-GAPDH antibody (Santa Cruz Biotechnology, USA), used for western blotting;

Rabbit secondary antibody, mouse secondary antibody (Biyuntian, China);

Anti-BANF1 antibody (Abcam, Cambridge), for Western blot analysis;

Anti-GAPDH antibody (Santa, USA), for Western blot analysis;

Anti-Ki67 antibody (ABclonal, China), for immunofluorescence staining;

Anti-cleaved-caspase3 antibody (Affinity), for both Western blot and immunofluorescence staining;

Anti-CCND1 antibody (Zhengneng Biotech, China), for Western blot analysis;

Anti-E-cadherin antibody (Proteintech, China), for Western blot analysis;

Anti-N-cadherin antibody (ImmunoWay, USA), for Western blot analysis;

Anti-Vimentin antibody (ImmunoWay, USA), for Western blot analysis;

Anti-Snai1 antibody (ImmunoWay, USA), for Western blot analysis;

Anti-BCL2 antibody (ImmunoWay, USA), for Western blot analysis;

Anti-caspase3 antibody (ABclonal, China), for Western blot analysis;

Anti-DNAPKcs antibody (Zhengneng Biotech, China), for Western blot analysis;

Anti-KU70 antibody (Zhengneng Biotech, China), for Western blot analysis;

Anti-KU80 antibody (Zhengneng Biotech, China), for Western blot analysis;

Anti-RAD51 antibody (ImmunoWay, USA), for Western blot analysis;

Rabbit secondary antibody, mouse secondary antibody (Biyuntian, China), for Western blot analysis.

Anti-BANF1 antibody (rabbit, Abcam, Cambridge), employed for Western blotting and immunofluorescence staining;

Anti-VRK1 antibody (rabbit, Immunoway, USA), used for Western blotting;

Anti-BANF1 antibody (rabbit, Thermo Fisher Scientific, USA), utilized for immunoprecipitation; Anti-VRK1 antibody (mouse, Santa Cruz Biotechnology, USA), applied in immunofluorescence staining and immunoprecipitation;

Mouse IgG Isotype Control (Zhengneng, China);
Rabbit IgG Isotype Control (Zhengneng, China).

Supplementary Table S3: The sequences of siRNAs.

si-RNA	sequences
si-GLI1	5′-CUCCACAGGCAUACAGGAU-3′
si-VRK1-1	5′-GCAGUUGGAGAGAUAAUAATT-3′
si-VRK1-2	5′-GCAGCUAAGCUUAAGAAUUTT-3′

Supplementary Table S4: Clinical information of patients with CRC.

id	age	sex	CEA	Stage	status	time	BANF1
P8	<65	female	>=5	III	0	1895	low
P2	<65	female	<5	II	1	1811	low
P39	<65	female	<5	II	0	2556	high
P17	<65	male	<5	IV	1	702	low
P30	<65	female	<5	II	0	2610	low
P41	<65	female	<5	II	0	2433	high
P48	<65	female	<5	III	1	728	high
P43	<65	female	>=5	II	0	2181	high
P40	<65	female	<5	II	0	2487	high
P67	<65	female	>=5	III	1	1770	high
P9	<65	male	<5	III	0	1559	low
P57	<65	female	>=5	II	1	356	high
P4	<65	male	>=5	II	0	2040	low
P16	<65	female	<5	III	1	386	low
P26	<65	female	<5	III	0	1852	low
P11	<65	male	<5	III	0	1284	low
P13	<65	female	<5	III	1	613	low
P54	<65	female	>=5	II	0	2050	high
P7	<65	male	>=5	III	0	2405	low
P1	<65	male	<5	II	0	2320	low
P34	<65	male	<5	II	1	2388	high
P24	<65	male	<5	III	0	1887	low
P27	<65	female	<5	I	0	2143	low
P23	<65	male	<5	II	0	1833	low
P62	<65	female	>=5	III	0	2027	high
P47	<65	male	>=5	III	1	333	high
P15	<65	male	<5	III	0	1980	low
P49	<65	male	<5	IV	1	1127	high
P60	<65	male	<5	III	0	2034	high
P61	<65	male	<5	III	0	2032	high
P10	<65	male	>=5	III	0	2402	low
P53	<65	female	>=5	IV	1	1464	high
P68	<65	female	>=5	IV	1	1006	high
P31	<65	female	<5	II	0	2610	low

Supplementary Table S4: Clinical information of patients with CRC.

P35	<65	female	<5	II	0	2310	high
P44	<65	male	>=5	II	0	2172	high
P58	<65	female	>=5	II	0	2029	high
P52	<65	female	>=5	IV	1	342	high
P28	<65	female	<5	I	0	2046	low
P33	<65	female	<5	II	1	2475	high
P56	<65	male	>=5	II	1	1890	high
P59	<65	male	<5	II	1	2014	high
P6	>=65	male	>=5	II	0	2309	low
P14	>=65	female	>=5	III	1	36	low
P37	>=65	male	<5	II	0	2110	high
P46	>=65	female	>=5	III	1	352	high
P25	>=65	male	<5	III	0	1863	low
P38	>=65	male	<5	II	1	2725	high
P3	>=65	female	<5	II	0	2173	low
P21	>=65	female	>=5	II	0	1734	low
P55	>=65	female	>=5	II	1	288	high
P18	>=65	male	<5	II	0	1910	low
P29	>=65	female	<5	I	1	1344	low
P5	>=65	female	>=5	II	0	2004	low
P42	>=65	female	<5	II	0	2223	high
P22	>=65	male	<5	II	1	129	low
P12	>=65	male	<5	III	0	1117	low
P32	>=65	male	<5	II	0	2543	low
P51	>=65	male	>=5	IV	1	69	high
P50	>=65	male	<5	IV	1	83	high
P63	>=65	male	>=5	III	1	1141	high
P65	>=65	female	>=5	III	1	630	high
P20	>=65	female	<5	II	0	1897	low
P64	>=65	male	>=5	III	1	83	high
P36	>=65	male	<5	II	1	2257	high
P45	>=65	female	>=5	II	1	1157	high
P66	>=65	male	>=5	III	1	708	high
P19	>=65	female	<5	II	1	2173	low
